# Rapid and reversible regulation of cell cycle progression in budding yeast using optogenetics

**DOI:** 10.1016/j.crmeth.2026.101541

**Published:** 2026-07-30

**Authors:** Andriana Koutsoumpa, Catherine Loukogiannaki, Danny Incarnato, Andreas Milias-Argeitis

**Affiliations:** 1 Molecular Systems Biology, Groningen Biomolecular Sciences & Biotechnology Institute, University of Groningen, Groningen, the Netherlands; 2 Molecular Genetics, Groningen Biomolecular Sciences & Biotechnology Institute, University of Groningen, Groningen, the Netherlands

**Keywords:** cell cycle, budding yeast, G1 cyclins, optogenetics, EL222, nutrient signaling, single-cell microscopy, cell cycle synchronization

## Abstract

The complexity of the eukaryotic cell cycle complicates experiment design and data interpretation, limiting our understanding of how cells coordinate cell cycle processes. Traditional perturbation methods, including knockouts, deletions, and arrest-inducing chemicals, are limited by compensatory feedback interactions or pleiotropic side effects. Inducible synthetic systems offer greater specificity but often rely on external inducers, making rapid reversibility difficult. Here, we developed OPTO-Cln2, an optogenetic tool for light-controlled and reversible regulation of G1 progression in budding yeast. Using time-lapse microscopy, we show that OPTO-Cln2-strains rapidly switch between normal and altered G1 progression. Combining OPTO-Cln2 with a readout of TORC1 and PKA activity, we find that oscillatory signaling dynamics is coordinated with G1 progression. Finally, we show that OPTO-Cln2 enables at least two cycles of synchronous arrest and release in liquid cultures. This system provides a powerful approach for studying cell cycle dynamics and the coordination of cell growth with division.

## Highlights

• OPTO-Cln2 enables reversible control of budding yeast G1 progression with blue light
• Light-induced arrest and release enables synchronization of yeast populations
• Nutrient signaling dynamics are tightly coordinated with G1 progression

## Motivation

Unraveling the mechanisms that coordinate cell growth with division requires tools that can perturb the cell cycle rapidly, reversibly, and with minimal side effects. Existing tools such as chemical inhibitors or inducible expression systems require medium exchange and are therefore difficult to scale for dynamic bulk-culture experiments. To address these challenges, we developed OPTO-Cln2, an optogenetic system that uses light to control G1 progression in budding yeast. As a simple, inexpensive and easily applied input, light allows OPTO-Cln2 to achieve non-invasive, reversible, and scalable control of cell cycle progression in both single-cell and bulk-culture studies.

## Graphical abstract

**Figure:**
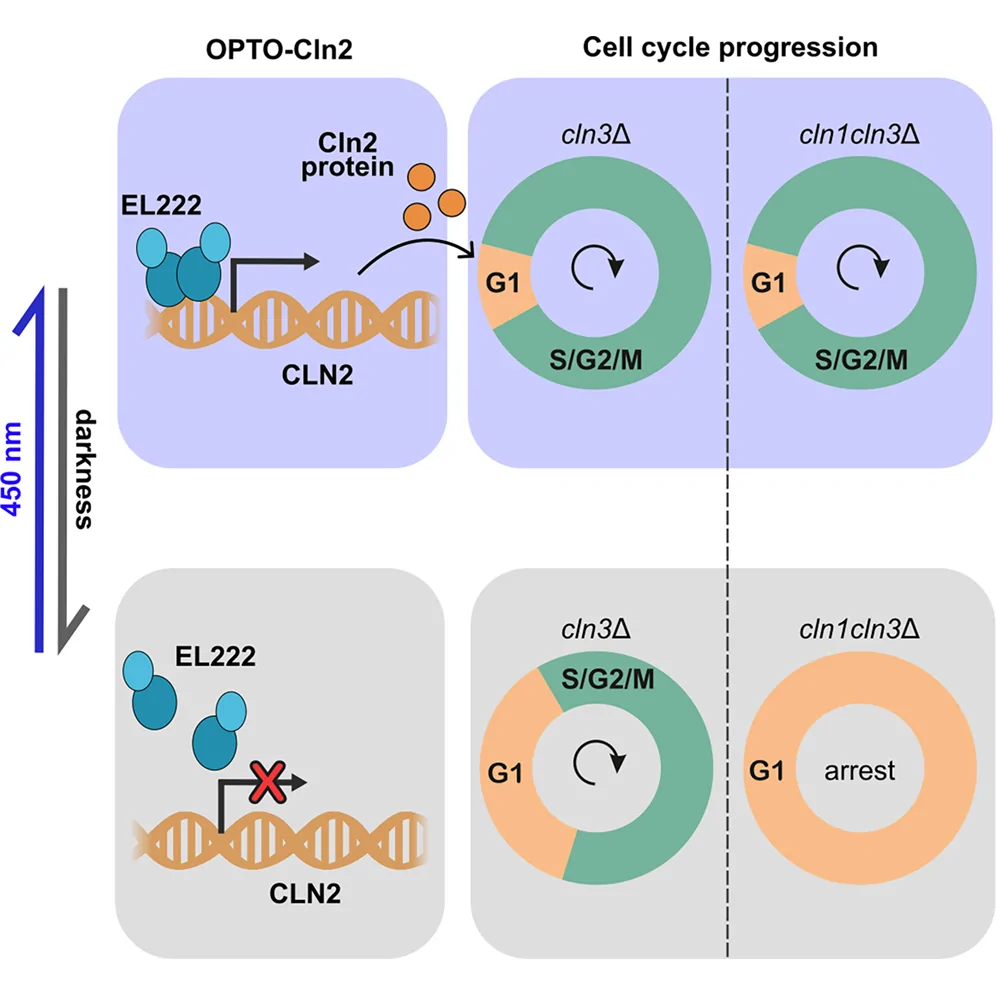

## Teaser

Koutsoumpa et al. present OPTO-Cln2, an optogenetic system for light-controlled *CLN2* expression in budding yeast based on an EL222-responsive promoter. In G1 cyclin mutant backgrounds, OPTO-Cln2 enables dynamic modulation of G1 progression and light-induced synchronization of cell populations.

## Introduction

The eukaryotic cell cycle comprises the processes by which cells grow, replicate their DNA, and divide.^1^ These processes are regulated in space and time through the coordinated action of signaling systems, transcriptional networks, and metabolic pathways. Despite significant decades of progress in the study of cell cycle control, complex feedback interactions among these regulators, together with multiple layers of redundancy, still pose major challenges for experimental design and data interpretation. As a result, our understanding of how cell cycle regulators interact dynamically and coordinate growth with division remains incomplete,^2^^,^^3^^,^^4^^,^^5^ even in unicellular eukaryotes such as budding yeast (*Saccharomyces cerevisiae*).^6^^,^^7^^,^^8^

Tools that perturb physiological cell cycle progression have been indispensable for the study of cell cycle control. Early approaches included deletions and mutations of key cell cycle regulators (e.g., temperature-sensitive mutants) and chemical agents that arrest the cell cycle.^9^ Although invaluable, these tools have important limitations.^10^ The effects of deletions and mutations can be masked by redundant pathways and compensatory feedback loops, blocking agents can trigger stress responses and reduce cell viability,^11^^,^^12^^,^^13^ and temperature-sensitive mutants are often hypoactive at the permissive temperature and retain residual function at non-permissive temperatures.^14^^,^^15^^,^^16^ In addition, shifting cells to non-physiological temperatures can itself induce stress responses.^17^

Analog-sensitive kinase technology provides another means to selectively inhibit protein kinases involved in cell cycle control.^18^ An analog-sensitive allele of the yeast CDK (Cdc28*as*) is available, but it exhibits substantially lower activity than the wild-type enzyme. Consequently, *cdc28-as* strains are larger and divide more slowly than the wild type.^19^ Moreover, arrest is not readily confined to a defined cell cycle stage, because addition of the ATP analog typically arrests cells at the cell cycle stage they occupy when CDK activity is inhibited.

Synthetic biology has enabled conditional knockout^20^ and overexpression^21^^,^^22^^,^^23^ systems that target specific cell cycle components. Together with chemicals that specifically inhibit cell cycle regulators,^23^^,^^24^^,^^25^ these tools allow precise and dynamic perturbation of cell cycle progression. A methionine-inducible Cln2 expression system has been used in several studies of cell cycle regulation and synchronization,^26^^,^^27^^,^^28^ while an estradiol-inducible Cln1 system was recently introduced.^29^^,^^30^^,^^31^ However, a key limitation is reversibility: switching these systems on and off typically requires adding and removing small-molecule inducers from the culture medium. Dynamic control of inducer levels often requires complex equipment, such as microfluidic or perfusion systems, and is difficult to scale to large cultures.

Optogenetics enables fast and targeted light-based modulation of protein activity,^32^ allowing reversible regulation of specific cell cycle components with high temporal resolution. Nevertheless, the application of optogenetics in cell cycle research has remained limited. Notable exceptions include an optogenetic system for light-inducible nuclear localization of the mammalian p21 cell cycle inhibitor^33^ and a system for light-controlled degradation of yeast Cdc48,^34^ a key regulator of ubiquitin-dependent protein degradation. Although loss of Cdc48 results in G2/M arrest, it also induces broad secondary effects reflecting the central role of the protein in multiple pathways.^35^ In another recent study on directed evolution in yeast, optogenetics was used to periodically overexpress cell cycle regulators (Cln2, Clb2, Clb5, and Cdc20).^36^ Since the primary aim of that study was to evolve protein function, the expression levels of these regulators were not optimized to minimize potential artifacts in cell morphology and/or cell cycle progression. To provide a minimally invasive alternative, we developed and characterized a transcriptional optogenetic system enabling precise regulation of G1 progression.

While deletion of all three G1 cyclins of yeast results in cell-cycle arrest,^37^ cells with a single G1 cyclin are viable with a prolonged G1 phase. Thus, it is possible to modulate G1 length or completely halt the cell cycle by controlling the expression of a G1 cyclin in a background strain lacking one or two G1 cyclins. To reversibly prolong or halt G1 progression, we developed the OPTO-Cln2 system. OPTO-Cln2 leverages the light-driven changes in the activity of the EL222 transcription factor^38^ to regulate the expression of the Cln2 G1 cyclin from an EL222-responsive promoter. In a *cln3Δ* background, OPTO-Cln2 enabled reversible switching between the wild-type and *cln2Δcln3Δ* phenotypes, toggling between fast and slow G1 progression. In a *cln1Δcln3Δ* background, it allowed reversible arrest and release of the cell cycle.

To validate our optogenetic strains, we used time-lapse microscopy to characterize their phenotypes and switching dynamics. Additionally, we compared the cell cycle progression and morphology of these strains to the corresponding features of *cln3Δ* and *cln2Δcln3Δ* strains, of which the latter had remained almost fully uncharacterized. We uncovered behaviors inconsistent with the established model of G1 regulation, suggesting compensatory mechanisms linked to cell size. Using a single-cell readout^39^ to monitor the cell cycle dynamics of two nutrient-sensing pathways (TORC1 and PKA) that regulate cell growth, we observed that their combined activity shows a maximum during G1 and minima at mitosis and budding (entry to S) under both light and dark conditions, suggesting that TORC1/PKA activity in G1 is coordinated with G1 progression. On the other hand, uncoupling growth from division in G1-arrested cells led to persistently high signaling activity, suggesting that entry to the S phase is accompanied by an attenuation of TORC1/PKA activity.

As a final demonstration of our optogenetic system, we monitored population dynamics during light-induced cell-cycle arrest and release. The OPTO-Cln2 system performed similarly to alpha factor treatment,^40^^,^^41^ the gold-standard arrest-and-release method for budding yeast. Unlike alpha factor, however, our approach requires no inducer addition or removal, enabling at least two arrest-release cycles in large culture volumes.

Optogenetic control of the cell cycle regulation can advance the study of cell cycle dynamics and enable new biotechnological applications. Because G1 progression mechanisms are conserved, our approach may be extended to higher eukaryotes, facilitating studies of medically relevant cell types such as stem cells and tumor cells.

## Results

### Deletion of G1 cyclins increases G1 duration in daughter cells and causes an increase in cell size

Since our optogenetic approach to cell cycle control is centered around the regulation of G1 progression, we first sought to establish the conditions under which this regulation can be effective and the phenotypes that can be attained under different light regimes. For this purpose, we first characterized the proliferation of yeast cells carrying G1 cyclin deletions.

Budding yeast cells missing one or two G1 cyclins are still able to progress through G1, whereas cells missing all three G1 cyclins arrest at the G1/S boundary.^37^ To determine how G1 cyclin deletion mutants differ from the prototrophic YSBN6 reference strain^42^ (henceforth abbreviated as “WT”) in terms of cell cycle progression and morphology, we constructed strains lacking one (*cln3Δ*) or two (*cln2Δcln3Δ*) G1 cyclins in a YSBN6 background carrying EL222 inserted at the HO locus (STAR Methods). Insertion of EL222 without its cognate light-responsive promoter did not affect cell proliferation (Figure S1). We therefore introduced EL222 into the deletion strains to ensure that all comparisons with the corresponding optogenetic strains were made in an equivalent genetic background.

Estimation of population doubling times via flow cytometry showed that the *cln2Δcln3Δ* mutant proliferated considerably slower than the WT (Figure S1). To observe in more detail how cell cycle progression and cell size are affected by G1 cyclin deletions, we used time-lapse microscopy to determine the distributions of G1 and post-G1 phase durations (roughly corresponding to the unbudded vs. the budded part of the cell cycle, STAR Methods), as well as the distributions of cell volumes at key cell cycle events (STAR Methods) in mother and daughter cells. The distinction between mothers and daughters was necessary because of their different size requirements for cell cycle commitment,^43^ which led to differences in cell cycle progression (Figure 1B). Full distributions of our measurement are provided in Figure 1, while Tables S1A–S1D summarize the distribution medians (which are also used throughout in the text) and Tables S1F–S1I provide statistical comparisons between different strains. These comparisons are also summarized graphically in Figure S2.

**Figure 1:**
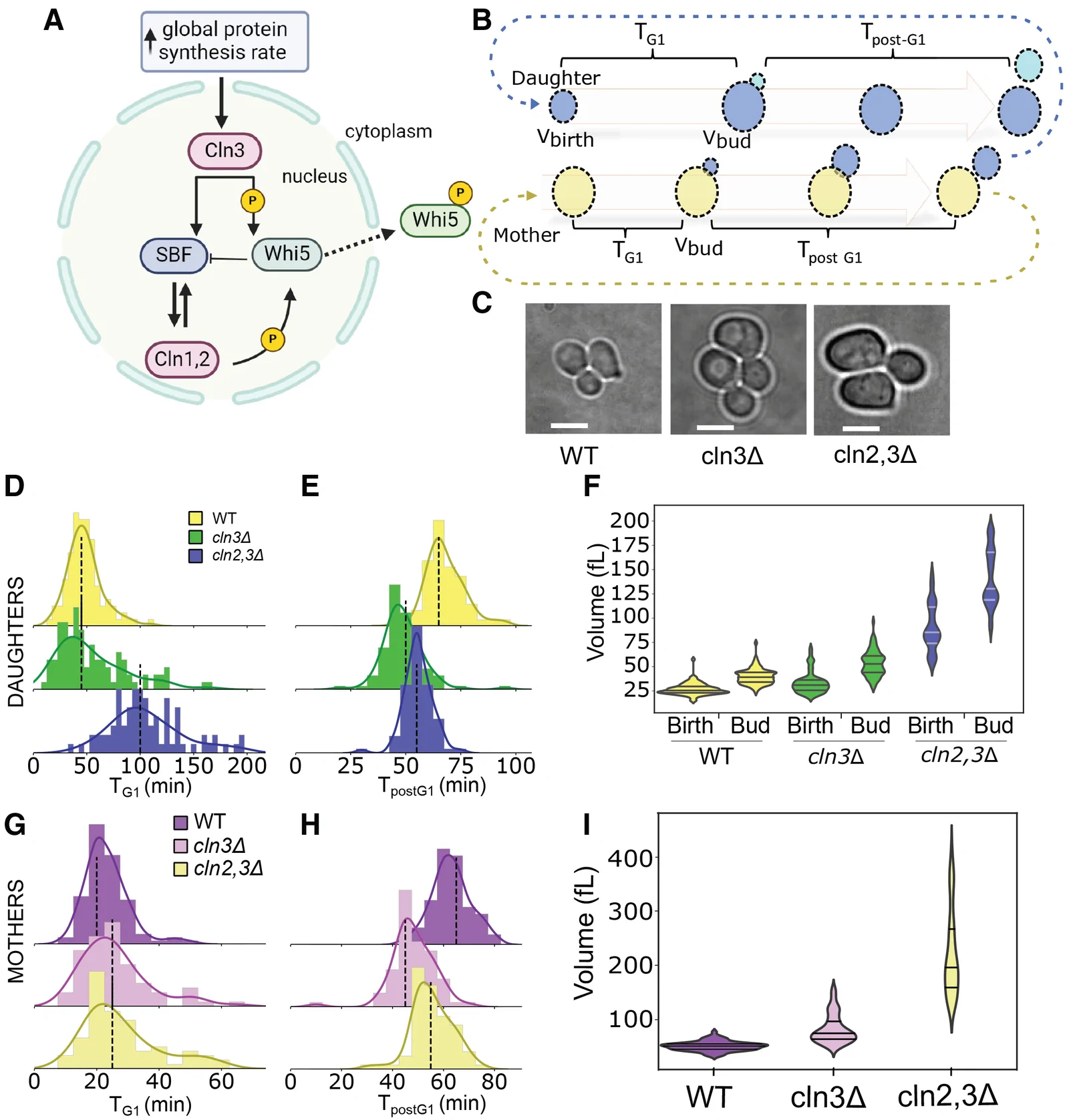
G1 cyclin mutants display differences in cell cycle progression and cell size (A) Schematic representation of the key players of the START transition network and their interactions. Cdk1 is the main CDK involved in cell cycle regulation in budding yeast.^44^ During G1, Cdk1 activity is driven by three G1 cyclins, Cln1, Cln2, and Cln3 (functionally analogous to mammalian cyclins D and E).^45^^,^^46^ An increase in the expression of Cln3^47^ leads to the formation of a Cln3/Cdk1 complex that eventually activates two major transcription factor complexes, SBF and MBF.^48^^,^^49^ These complexes drive the transcription of Cln1 and Cln2, as well as hundreds of additional genes required for the G1/S transition.^50^^,^^51^ Expression of Cln1 and Cln2 sets up a positive feedback loop in which Cln1/Cdk1 and Cln2/Cdk1 complexes phosphorylate and deactivate Whi5, a transcriptional repressor that binds and inhibits SBF. Deactivation of Whi5 triggers the G1/S transition and bud formation.^52^^,^^53^ (B) Illustration of the experimentally determined cell cycle phase durations and cell volumes for mother and daughter cells. (C) Representative bright-field images of WT, *cln3Δ*, and *cln2,3Δ* cells. Scale bars, 5 μm. (D) Distributions of G1 durations (T_G1_) in WT (*n* = 109), *cln3Δ* (*n* = 97), and *cln2,3Δ* (*n* = 79) daughter cells. Medians are indicated with dashed lines: WT = 45 min, *cln3Δ* = 45 min, *cln2,3Δ* = 100 min. (E) Distributions of post-G1 durations (T_postG1_) in WT (*n* = 109), *cln3Δ* (*n* = 97), and *cln2,3Δ* (*n* = 79) daughter cells. Medians are indicated with dashed lines: WT = 65 min, *cln3Δ* = 50 min, *cln2,3Δ* = 55 min. (F) Volume distributions at birth (V_birth_) and budding (V_bud_) for WT (*n* = 82), *cln3Δ* (*n* = 77), and *cln2,3Δ* (*n* = 44) daughter cells. Medians: WT_birth_ = 25.01 fl, WT_bud_ = 38.76 fl, *cln3Δ*_birth_ = 31.62 fl, *cln3Δ*_bud_ = 52.45 fl, *cln2,3Δ*_birth_ = 85.07 fl, *cln2,3Δ*_bud_ = 129.99 fl. The medians along with the 25th and 75th percentiles are also provided in Table S1A. (G) Distributions of G1 durations (T_G1_) in WT (*n* = 97), *cln3Δ* (*n* = 71), and *cln2,3Δ* (*n* = 75) mother cells. Medians are indicated with dashed lines: WT = 20 min, *cln3Δ* = 25 min, *cln2,3Δ* = 25 min. (H) Distributions of post-G1 durations (T_postG1_) in WT (*n* = 97), *cln3Δ* (*n* = 71), and *cln2,3Δ* (*n* = 75) mother cells. Medians are indicated with dashed lines: WT = 65 min, *cln3Δ* = 45 min, *cln2,3Δ* = 55 min. (I) Volume distributions at budding for WT (*n* = 70), *cln3Δ* (*n* = 76), and *cln2,3Δ* (*n* = 46) mother cells. Medians: WT_bud_ = 52.59 fl, *cln3Δ*_bud_ = 76.09 fl, *cln2,3Δ*_bud_ = 201.97 fl. The medians along with the 25th and 75th percentiles are also provided in Table S1C.

Collectively, our results showed that, while loss of Cln3 does not lead to any pronounced changes in G1 duration in mothers or daughters, the loss of both Cln2 and Cln3 has a substantial impact on the G1 duration of daughters (Figures 1D–1G). Moreover, loss of G1 cyclins tends to cause a slight shortening of post-G1 phase durations in both mothers and daughters (Figures 1E–1H), with the effect being more pronounced in *cln3Δ* cells. Deletions of G1 cyclins result in cells that are larger than the WT as previously observed,^50^^,^^54^ with the double cyclin deletion resulting in the largest volume increase (Figures 1F–1I). These observations will be used in the development of our optogenetic systems below.

### An optogenetic system for light-driven Cln2 expression: General design considerations

Our study of G1 cyclin deletion mutants showed that the transition from two functional cyclins to one causes a significant delay in daughter G1 progression. On the other hand, it is well-established that loss of the remaining cyclin leads to cell-cycle arrest. It should, therefore, be possible to construct optogenetic systems that can either slow down or completely halt the cell cycle. To achieve these effects, one can conditionally express a G1 cyclin in a background where either one or two other G1 cyclins are deleted. Conditionally expressing any of the three G1 cyclins should be possible in theory, but we chose Cln2 because it has been found to exert stronger control on G1 progression than Cln1.^55^

To regulate Cln2 transcription in a light-dependent manner, we used a previously developed optogenetic construct^56^ that consists of the well-studied light-sensitive EL222 transcription factor^38^^,^^57^^,^^58^^,^^59^ fused to the VP16 transcriptional activation domain. Since the wild-type EL222 protein has a dark reversion time of 1–2 min^38^ and Cln2 is an unstable protein^60^ (τ_1/2_ ∼10–15 min), sustained Cln2 expression over several minutes would require nearly continuous illumination, which would, in turn, limit the number of image fields that can be simultaneously stimulated and observed in microscopy experiments. We therefore used an EL222 mutant (EL222-AQTrip^59^) that has a slower reversion time (∼30 min). The slow reversion of this mutant allows the use of brief (∼1 s) light pulses every 5 min for establishing and maintaining sufficiently high Cln2 expression levels across a cell population. To maintain as uniform EL222 levels as possible, the VP16:EL222-AQTrip construct was integrated into the HO locus of our background strain, under the control of the constitutive ACT1 promoter.

Once activated by low-intensity blue (∼450–470 nm) light, EL222 homodimerizes and binds to its cognate binding site (called C120 in Motta-Mena et al.^38^) with very high specificity.^38^^,^^58^ Insertion of multiple EL222-binding sites to the promoter region upstream of a given target gene ensures sufficiently strong and uniform activation by EL222.^56^ Cln2 is a weakly expressed protein,^61^ and it is well-established that Cln2 overexpression causes severe morphological artifacts (elongated, non-symmetric cells with an aberrant budding pattern) and slow proliferation.^62^^,^^63^^,^^64^ To avoid these artifacts while maintaining cell cycle control, an inducible Cln2 expression system would need to combine low maximal activity in the light (to keep Cln2 levels within physiological limits) with minimal leakage in the dark (to ensure Cln2 cannot promote cell cycle commitment without light stimulation).

To identify a promoter with the desired features, we considered a recently presented collection of EL222-responsive promoters for budding yeast.^56^ Focusing on the two promoters at the low end of the expression scale, we chose the *pSPO13* and *pGAL1* backbones for further testing and introduced them at the endogenous *CLN2* locus by replacing the 700 bp promoter region upstream of the *CLN2* open reading frame (ORF). Spo13 is a meiotic regulator whose expression is tightly repressed during vegetative growth,^65^ while Gal1 expression is tightly repressed under glucose-rich conditions.^66^ Despite this fact, cells harboring the *pGAL1*-based EL222 promoter showed signs of Cln2 overexpression (elongated shape and slow proliferation, Figure S3A) even in darkness, suggesting that the leakage of this promoter is too high even under repressing conditions. On the other hand, we did not observe any signs of Cln2 overexpression in cells carrying the *pSPO13*-based promoter at the endogenous *CLN2* locus. Interestingly, deletion of the endogenous *CLN2* gene and introduction of a pSPO13-driven copy of *CLN2* in the X-1 integration locus^67^ resulted in Cln2 overexpression (Figures S3D and S3E), demonstrating that the choice of integration locus can affect protein levels. Moreover, a longer (∼1,000-bp) truncation of the endogenous *CLN2* promoter was also found to be suboptimal, as it resulted in enlarged cells with highly variable daughter G1 durations (Figure S3F).

Based on these results, the optogenetic strains presented below carried the *pSPO13*-based promoter inserted at the endogenous *CLN2* locus, combined with a 700-bp truncation of the endogenous *CLN2* promoter. Henceforth, we will refer to this light-dependent *CLN2* construct as the OPTO-Cln2 system (Figure 2A).

**Figure 2:**
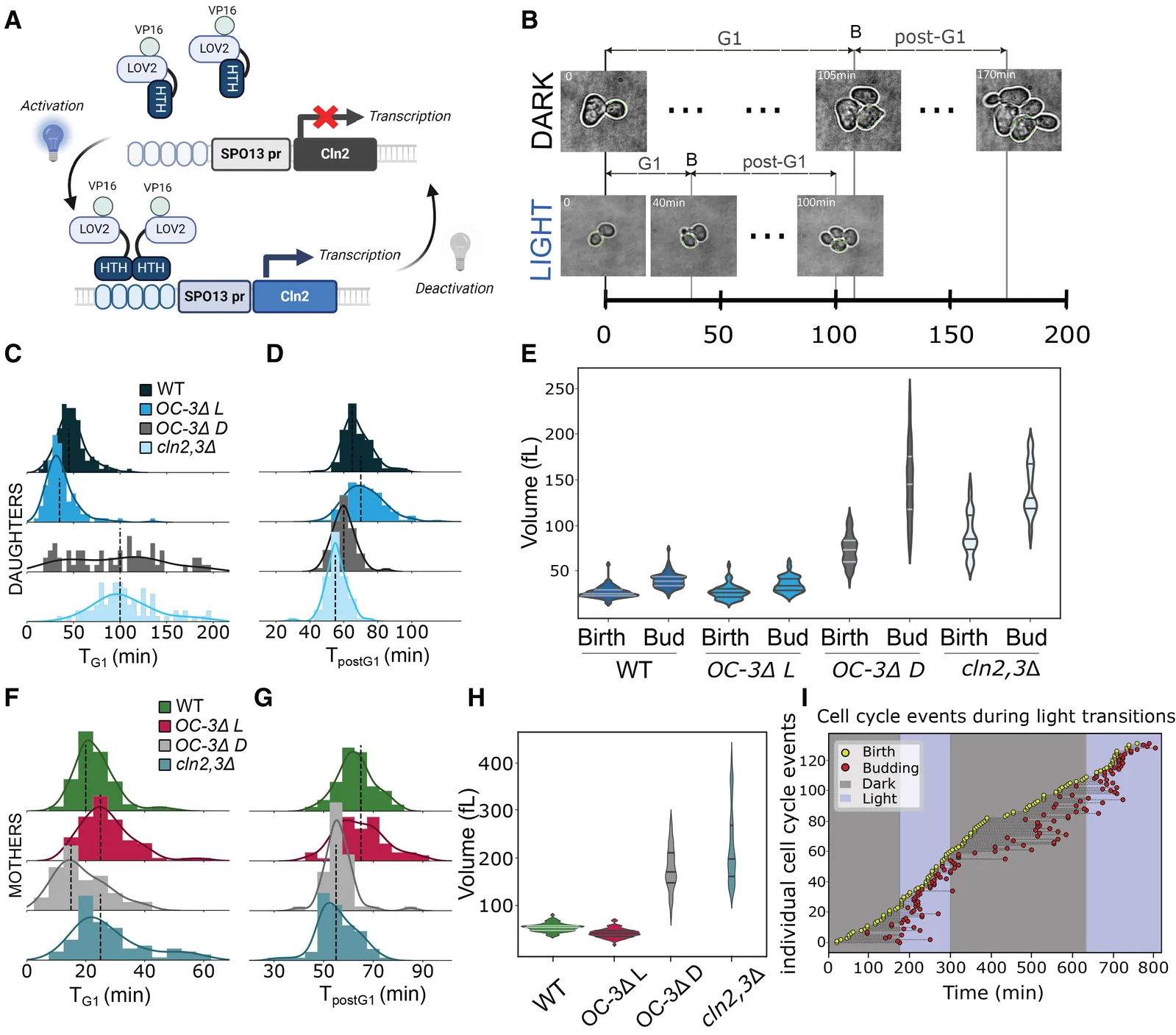
The OPTO-Cln2 system and dynamic regulation of G1 progression in an OPTO-Cln2 *cln3Δ* background (A) Activation of VP16-EL222 by low-intensity blue light triggers EL222 homodimerization and binding to its cognate binding sites (light-blue ovals in front of the promoter). These sites are introduced upstream of the *SPO13* promoter, which replaces the endogenous *CLN2* promoter. Exposure to blue light activates *CLN2* transcription within a few minutes.^68^ In the absence of light, EL222 spontaneously reverses to its dark state and is released from DNA, shutting down *CLN2* transcription. The EL222 variant used here (AQTrip) has a dark reversion half-time of ∼30 min.^59^ (B) Phenotypic differences of OPTO-Cln2 daughter cells in light vs. dark conditions. Light activation leads to accelerated G1 progression and smaller cell volumes, while darkness increases both G1 duration and cell volumes. (C) Distributions of G1 durations (T_G1_) in daughters of WT (*n* = 109), the OPTO-Cln2 *cln3Δ* (denoted OC-3Δ) strain in light (OC-3Δ L; *n* = 106) and darkness (OC-3Δ D; *n* = 61), and *cln2,3Δ* (*n* = 79). Medians are indicated with dashed lines: WT = 45 min, OC-3Δ L = 35 min, OC-3Δ D = 100 min, *cln2,3Δ* = 100 min. (D) Distributions of post-G1 durations (T_postG1_) in WT (*n* = 109), OC-3Δ L (*n* = 106), OC-3Δ D (*n* = 61), and *cln2,3Δ* (*n* = 79) daughters. Medians are indicated with dashed lines: WT = 65 min, OC-3Δ L = 70 min, OC-3Δ D = 60 min, *cln2,3Δ* = 55 min. (E) Volume distributions of daughter cells at birth (V_birth_) and budding (V_bud_) for WT (*n* = 82), OC-3Δ L (*n* = 63), OC-3Δ D (*n* = 104), and *cln2,3Δ* (*n* = 44). Medians: WT_birth_ = 25.01 fl, WT_bud_ = 38.76 fl, OC-3Δ L_birth_ = 26.18 fl, OC-3Δ L_bud_ = 33.71 fl, OC-3Δ D_birth_ = 73.23 fl, OC-3Δ D_bud_ = 145.22 fl, *cln2,3Δ*_birth_ = 85.07 fl, *cln2,3Δ*_bud_ = 129.99 fl. The medians, along with the 25th and 75th percentiles, are displayed with continuous lines and are also provided in Table S1A. (F) Distributions of G1 durations (T_G1_) in WT (*n* = 97), OC-3Δ L (*n* = 75), OC-3Δ D (*n* = 51), and *cln2,3Δ* (*n* = 75) mother cells. Medians are indicated with dashed lines: WT = 20 min, OC-3Δ L = 25 min, OC-3Δ D = 15 min, *cln2,3Δ* = 25 min. (G) Distributions of post-G1 durations (T_postG1_) in WT (*n* = 97), OC-3Δ L (*n* = 75), OC-3Δ D (*n* = 51), and *cln2,3Δ* (*n* = 75) mothers. Medians are indicated with dashed lines: WT = 65 min, OC-3Δ L = 65 min, OC-3Δ D = 55 min, *cln2,3Δ* = 55 min. (H) Volume distributions in mother cells at budding for WT (*n* = 75), OC-3Δ L (*n* = 70), OC-3Δ D (*n* = 63), and *cln2,3Δ* (*n* = 46). Medians: WT_bud_ = 52.59 fl, OC-3Δ L_bud_ = 40.12 fl, OC-3Δ D_bud_ = 169.83 fl, *cln2,3Δ*_bud_ = 201.97 fl. The medians along with the 25th and 75th percentiles are also provided in Table S1C. (I) Cell cycle events of individual OC-3Δ daughters during light transitions. Cells were annotated for the moments of birth (yellow circles) and budding (red circles). In this way, the length of the dashed line segment between birth and budding corresponds to the G1 duration. Gray and blue segments denote intervals of darkness and light exposure, respectively.

### The OPTO-Cln2 system enables dynamic control of G1 progression

Based on our characterization of the G1 cyclin-deficient strains presented above, we first built a strain capable of transitioning between fast and slow cell cycle progression. To achieve this, we put Cln2 expression under light control in a strain lacking *CLN3*. Upon light exposure, we expected that this strain should divide as fast or faster than the WT due to continuous Cln2 synthesis, which bypasses the requirement for Cln3 in early G1. Conversely, subjecting the cells to darkness should result in EL222 deactivation and Cln2 degradation, leaving Cln1 as the sole activator of the G1/S transition. This condition was expected to induce a phenotype resembling the *cln2Δcln3Δ* mutant.

Liquid cultures of OPTO-Cln2 *cln3Δ* cells had a population doubling time of 115 min under exponential growth in the dark, which is between the 85 min of the WT strain and the 139 min of the *cln2Δcln3Δ* mutant (Figure S1). On the other hand, light-exposed cultures of OPTO-Cln2 *cln3Δ* cells displayed a population doubling time of 81 min, which is slightly shorter than the WT doubling time.

To determine in detail how the OPTO-Cln2 *cln3Δ* cells are affected by the presence or absence of light, we tracked the durations of G1 and non-G1 cell cycle phases of these cells with time-lapse microscopy, using WT and the *cln2Δcln3Δ* mutant as reference points. Dark-grown OPTO-Cln2 *cln3Δ* daughter cells had a prolonged G1 phase (100 min), similar to the *cln2Δcln3Δ* mutant and considerably longer than the average G1 duration of WT daughters (45 min) (Figure 2C). Growth under low-intensity blue light caused a massive decrease in G1 phase duration of OPTO-Cln2 *cln3Δ* daughters (35 min), which now entered the cell cycle much sooner than the WT daughters. On the other hand, the post-G1 duration of dark-grown OPTO-Cln2 *cln3Δ* daughters (60 min) was between that of the *cln2Δcln3Δ* mutant (55 min) and the WT cells (65 min) and increased slightly (70 min) when these daughters were grown under blue light (Figure 2D). Overall, the light-induced changes in daughter cell cycle progression indicated that the OPTO-Cln2 system in a *cln3Δ* background can strongly modulate G1 duration, producing daughters that divide as slow as *cln2Δcln3Δ* or faster than WT.

Dark-grown OPTO-Cln2 *cln3Δ* mother cells did not display major changes between light and darkness, either in G1 (25 vs. 15 min) or in post-G1 duration (55 vs. 65 min) (Figures 2F and 2G). These phase durations were close to those of WT (G1: 20 min, post-G1: 65 min) and *cln2Δcln3Δ* cells (G1: 25 min, post-G1 55 min). Therefore, the OPTO-Cln2 system does not seem to affect cell cycle progression in OPTO-Cln2 *cln3Δ* mothers, as was already expected from the fact that WT and *cln2Δcln3Δ* mothers have similar cell cycle statistics.

As discussed above, besides affecting cell cycle progression, G1 cyclin deletions also affect the average sizes of both daughter and mother cells. To investigate whether the effect of the OPTO-Cln2 system induces cell volume changes in line with those expected from our results above, we quantified volumes of OPTO-Cln2 *cln3*Δ mothers at budding and daughter volumes at birth and budding, using the WT and *cln2Δcln3Δ* strains as reference points for light and dark conditions, respectively. Light-grown OPTO-Cln2 *cln3Δ* daughters resembled WT daughters in terms of volume (birth: 26 fl vs. 25 fl, budding: 34 fl vs. 39 fl) (Figure 2E). On the other hand, dark-grown OPTO-Cln2 *cln3Δ* daughters displayed a more than 2-fold increase in volume compared to WT (birth:73 fl, budding: 145 fl), in good agreement with the average volumes of *cln2Δcln3Δ* daughters (birth: 85 fl, budding: 130 fl). Light-grown OPTO-Cln2 *cln3Δ* mother cells were slightly smaller than WT at budding (40 fl vs. 53 fl), while growth in darkness increased the average volume (170 fl) close to that of the *cln2Δcln3Δ* reference strain (201 fl) (Figure 2H).

Taken together, our results indicate that the OPTO-Cln2 system in a *cln3Δ* background reproduces the characteristic cell cycle and volume phenotypes of the *cln2Δcln3Δ* mutant in the dark. In the light, the system produces cells that are slightly smaller and divide faster than the WT, consistent with the notion that constitutive Cln2 expression compensates for the absence of Cln3 and promotes an earlier transition into S phase. To examine how these phenotypes relate to *CLN2* expression levels in darkness vs. light, we quantified CLN2 mRNA by quantitative reverse-transcription PCR (RT-qPCR) (Figures S4A and S4B). Consistent with our phenotypic observations, *CLN2* expression in OPTO-Cln2 *cln3Δ* cells decreased to approximately 25% of the WT levels in darkness, but reached WT levels under illumination. Residual *CLN2* expression in darkness, likely from promoter leakage and/or residual EL222 activity, accounts for the slightly less severe phenotype of this strain compared to *cln2Δcln3Δ*. Although the RT-qPCR results are consistent with the observed phenotypes, direct measurements of Cln2 protein levels could provide additional insight into the relationship between Cln2 expression and cell cycle dynamics. However, given the low abundance and rapid turnover of Cln2, accurate protein quantification remains technically challenging and was, therefore, beyond the scope of the present study.

In summary, our tests demonstrated that the OPTO-Cln2 *cln3Δ* daughters can switch between a long and a short cell cycle driven by light-induced changes in *CLN2* expression and that this effect is mostly attributed to the acceleration or slowdown of G1 phase (Figure 2B). On the other hand, the cell cycle of mother cells remains largely unaltered in this optogenetic strain.

Following the characterization of the OPTO-Cln2 *cln3Δ* strain under steady-state growth, we tested how quickly cells respond to changes in light conditions. For this purpose, we used time-lapse microscopy to monitor the cell cycle progression of daughter cells, since this is the population that is affected by the light input. Our results revealed that daughters born shortly before or right after a darkness-to-light transition, exhibited a shorter G1 phase compared to daughters born in darkness (Figure 2I). Conversely, daughters born shortly after a transition from light to darkness displayed a longer G1 compared to daughters born in light. These findings suggest that Cln2 levels respond fast to light transitions and that this response drives changes in the G1 duration for daughters that are born close to the transition times. In summary, the OPTO-Cln2 *cln3Δ* strain switches between fast- and slow-dividing daughter phenotypes, with fast transitions in either direction.

### The OPTO-Cln2 system generates light-dependent arrest and release

To develop a strain that can divide only in the presence of blue light, we introduced OPTO-Cln2 in a *cln1Δcln3Δ* background. Contrary to the slow-dividing OPTO-Cln2 *cln3Δ* daughters, cell cycle progression of OPTO-Cln2 *cln1Δcln3Δ* is expected to be halted at the G1/S boundary when daughter and mother cells are shifted to darkness.

To determine if this strain undergoes arrest in the absence of light, a liquid culture growing in the presence of blue light was shifted to darkness, while cell numbers were continuously monitored using flow cytometry. Following the shift to darkness, cell proliferation appeared to come to a halt within 1.5–2 h, as was evident from the stabilization of cell counts (Figure S4D). This delay is expected since cells need to complete the cycle in which the light transition occurred and arrest in the G1 phase of the following cycle. This observation implies that Cln2 expression quickly drops below the threshold required to support proliferation when cells are moved to darkness.

A more detailed characterization of this strain via time-lapse microscopy showed that the average G1 duration of daughter cells grown under blue light was around the same as the average G1 duration of the WT cells (40 min vs. 45 min), consistent with the fact that the presence of Cln2 bypasses the need for Cln3 upregulation in early G1 (Figure 3A). Rapid passage through G1 was also evident in the volumes of these daughter cells, which budded at a smaller size compared to WT (27 fl vs. 38 fl) (Figure 3C). A moderate but significant increase was observed in the post-G1 phase duration of these light-grown cells compared to WT (75 min vs. 65 min) (Figure 3B).

**Figure 3:**
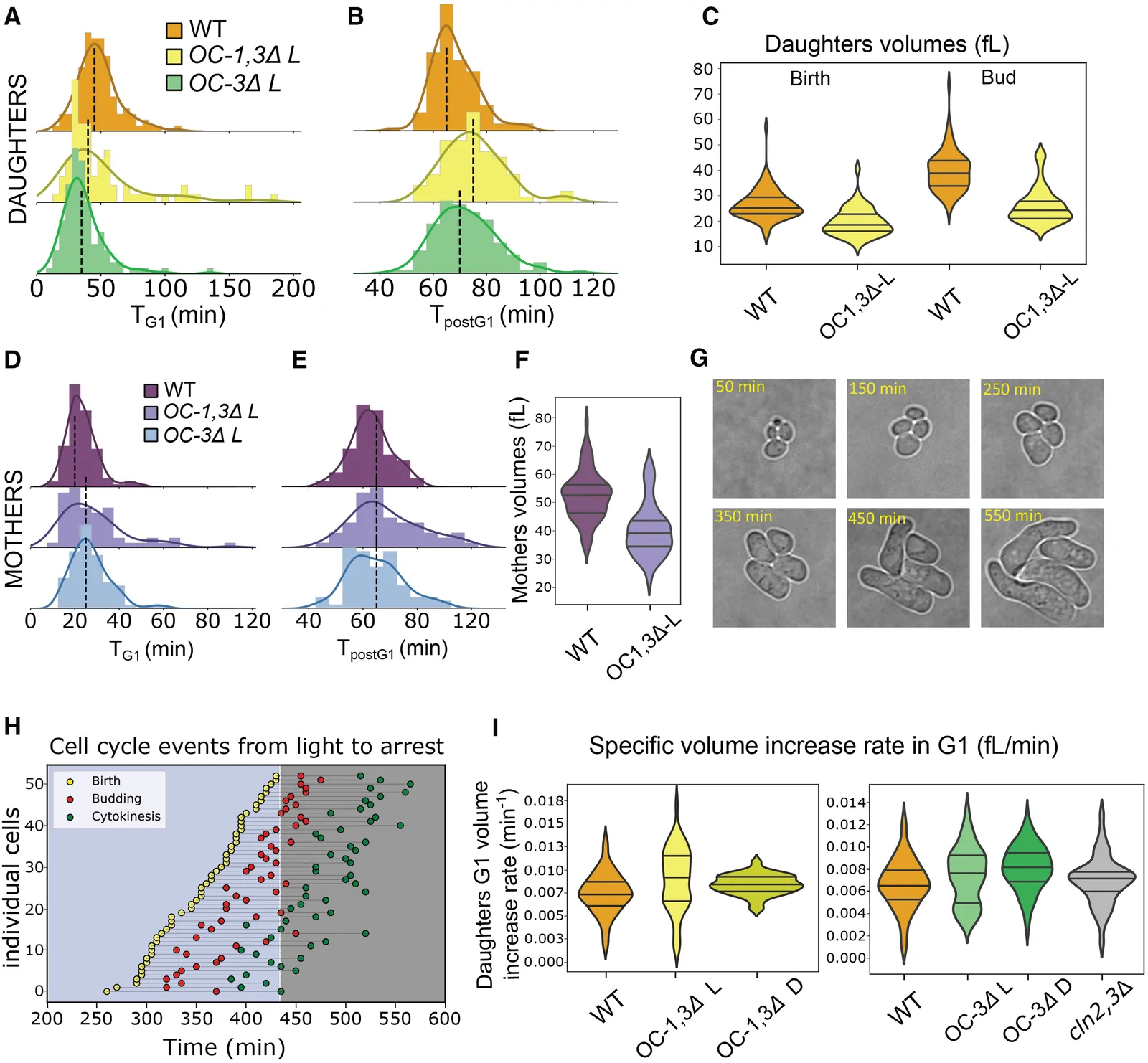
Dynamic regulation of cell-cycle arrest and release with the OPTO-Cln2 system (A) Distributions of G1 durations (T_G1_) in WT (*n* = 109), OC-3Δ L (*n* = 72), and OPTO-Cln2 *cln1*Δ*cln3*Δ (denoted OC-1,3Δ) in light (OC-1,3Δ L; *n* = 106) daughters. Medians are indicated with dashed lines: WT = 45 min, OC-1,3Δ L = 40 min, OC-3Δ L = 35 min. Statistical comparisons, two-tailed Mann-Whitney test: WT − OC-1,3Δ *p* = 0.12, |r| = 0.13; OC-3Δ − OC-1,3Δ, *p* = 9e-4, |r| = 0.29. (B) Distributions of post-G1 durations (T_postG1_) in WT (*n* = 109), OC-3Δ L (*n* = 72), and OC-1,3Δ L (*n* = 106) daughters. Medians are indicated with dashed lines: WT = 65 min, OC-1,3Δ L = 75 min, OC-3Δ L = 70 min. Statistical comparisons, two-tailed Mann-Whitney test: WT − OC-1,3Δ, *p* = 7re-6, |r| = 0.39; OC-3Δ − OC-1,3Δ, *p* = 0.09, |r| = 0.12. (C) Volume distributions of daughter cells at birth and budding for WT (*n* = 82) and OC-1,3Δ L (*n* = 48). Medians: WT_birth_ = 25.01 fl, WT_bud_ = 38.76 fl, OC-1,3Δ L_birth_ = 18.3 fl, OC-1,3Δ L_bud_ = 24.1 fl. The medians along with the 25th and 75th percentiles are also provided in Table S1A. Statistical comparisons, two-tailed Mann-Whitney test: WT(birth) − OC-1,3Δ(birth), *p* = 4.47e-10, |r| = 0.66; WT(bud) − OC-1,3Δ(bud) *p* = 4.6e-14, |r| = 0.79. (D) Distributions of G1 durations (T_G1_) in WT (*n* = 97), OC-3Δ L (*n* = 75), and OC-1,3Δ L (*n* = 57) mothers. Medians are indicated with dashed lines: WT = 20 min, OC-1,3Δ L = 25 min, OC-3Δ L = 25 min. Statistical comparisons, two-tailed Mann-Whitney test: WT − OC-1,3Δ, *p* = 0.04, |r| = 0.19; OC-3Δ − OC-1,3Δ, *p* = 0.85, |r| = 0.02. (E) Distributions of post-G1 durations (T_postG1_) in WT (*n* = 97), OC-3Δ L (*n* = 75), and OC-1,3Δ L (*n* = 57) mothers. Medians are indicated with dashed lines: WT = 65 min, OC-1,3Δ L = 65 min, OC-3Δ L = 65 min. Statistical comparison, two-tailed Mann-Whitney test: WT − OC-1,3Δ, *p* = 0.008, |r| = 0.25; OC-3Δ − OC-1,3Δ, *p* = 0.09, |r| = 0.17. (F) Volume distributions of mother cells at budding for WT (*n* = 70) and OC-1,3Δ L (*n* = 38). Medians: WT_bud_ = 52.59 fl, OC-1,3Δ L_bud_ = 39.07 fl. The medians along with the 25th and 75th percentiles are also provided in Table S1C. Statistical comparison, two-tailed Mann-Whitney test: WT(bud) − OC-1,3Δ(bud), *p* = 1.3e-8, |r| = 0.66. (G) Bright-field images of OC-1,3Δ light-grown cells, shifted to darkness at t = 0 and grown under YNB + glucose 2% agar pads for several hours. (H) Cell cycle events of individual OC-1,3Δ daughters during light transitions. Cells were tracked for ∼8 h during a transition from light to darkness, and the cell cycle events (birth, budding, and cytokinesis) of the last cycle before the arrest were annotated. Daughters that were born within this interval but did not bud until the end of the experiment were excluded from the plot. Figure S4C displays all tracked daughters of that experiment, including the ones that did not bud. (I) Distributions of specific volume increase rates during G1 in daughters of WT (*n* = 82), OC-1,3Δ D (*n* = 64) and OC-1,3Δ L (*n* = 47), OC-3Δ D (*n* = 103) and OC-3Δ L (*n* = 57), and *cln2,3Δ* (*n* = 44). Medians: WT = 0.0065 min^−1^, OC-1,3Δ D = 0.0070 min^−1^, OC-1,3 L = 0.0083 min^−1^, OC-3Δ L = 0.0076 min^−1^, OC-3Δ D = 0.0081 min^−1^, *cln2,3Δ* = 0.0072 min^−1^. The medians along with the 25th and 75th percentiles are also provided in Table S1E. The WT distribution is presented twice to facilitate comparisons. Statistical comparisons are provided in Table S1J and are summarized graphically in Figure S2C.

When grown under blue light, mother cells of the OPTO-Cln2 *cln1Δcln3Δ* strain displayed G1 and post-G1 phase durations that were similar to those of WT mothers (25 min vs. 20 min for G1, 65 min for post-G1) (Figures 3D and 3E). Moreover, the volumes of OPTO-mother cells were smaller compared to WT mothers (39 fl vs. 53 fl) (Figure 3F). In brief, our results on light-grown OPTO-Cln2 *cln1Δcln3Δ* cells suggest that, in the presence of light, these cells proliferate similarly to light-grown OPTO-Cln2 *cln3Δ* and faster than WT cells, thanks to the shortened G1 duration of daughters.

To further confirm that cell cycle progression proceeds normally under light induction, we monitored Whi5-mCherry localization in light-grown OPTO-Cln2 *cln1Δcln3Δ* cells. As expected, Whi5 accumulates in the nucleus during late mitosis and early G1 and moves to the cytoplasm at budding, both in mothers and daughters (Figure S5A). This observation confirms that the regulatory link between Whi5 localization and G1 progression is preserved in this optogenetic strain.

When transferred to darkness, OPTO-Cln2 *cln1Δcln3Δ* cells arrest in the unbudded state (Figure 3G). Moreover, the transition from proliferation to arrest typically occurs within a single cell cycle. Figure 3H summarizes the dynamics of the light-to-darkness transition of daughter cells tracked by time-lapse microscopy. The light switch occurs 440 min after the start of the experiment and is indicated by the change in background shading. Cell traces are ordered according to the timing of daughter birth (yellow circles). For each cell, the moments of budding and cytokinesis are marked by red and green circles, respectively, and traces terminate when no further budding events are observed. As Figure 3H shows, all tracked cells eventually arrest in darkness. Daughters that experience the light transition during G1 occasionally initiate one additional budding event in darkness, whereas those that have already budded before the light transition do not divide again once the light is turned off. Consistent with G1 arrest, Whi5-mCherry accumulates in the nucleus during the transition from proliferation to arrest and exits upon re-illumination (Figure S5B).

We observed that the arrested cells continued to grow in volume and acquired irregular shapes, while prolonged arrest (>8 h) led to cell death (Figure 3G). This decoupling of volume dynamics from cell cycle activity prompted us to investigate more broadly whether the specific volume increase rate of OPTO-Cln2 daughters during G1 differs from the corresponding rate of WT and *cln2,3Δ* daughters. WT and the *cln2,3Δ* mutant displayed a small but non-significant difference in their specific volume increase rates during G1 (0.0065 min^−1^ vs. 0.0072 min^−1^) (Figure 3I). On the other hand, the specific volume increase rates of OPTO-Cln2 *cln1Δcln3Δ* daughters (in both light and darkness) displayed minor but statistically significant increases compared to WT daughters (dark-grown vs. WT: 0.0071 min^−1^ vs. 0.0065 min^−1^, light-grown vs. WT: 0.0084 min^−1^ vs. 0.0065 min^−1^) (Table S1J; Figure S2C). Similarly, a minor but significant increase was observed in dark-grown OPTO-Cln2 *cln3Δ* daughters (0.0082 min^−1^) compared to WT and *cln2,3Δ* (Figure 3I). In summary, OPTO-Cln2 *cln1Δcln3Δ* daughters grow slightly faster compared to WT during G1, while OPTO-Cln2 *cln3Δ* daughters show a small increase in growth rate only in darkness. Given the large differences in G1 progression among these strains, the observed differences in growth rates are quite small, suggesting that the mechanisms regulating volume changes during G1 operate largely independently of the cell cycle machinery.

### Differential regulation of G1 cyclins in OPTO-Cln2 strains alters TORC1/PKA activity dynamics

Proliferating cells need to accumulate biomass and increase in volume before dividing. In budding yeast, the cellular processes that lead to biomass increase are tightly regulated by highly conserved signaling pathways centered around the target of rapamycin complex 1 (TORC1)^69^ and protein kinase A (PKA).^70^ These pathways respond to the availability, uptake, and utilization of nutrients to balance anabolic processes that increase biomass with catabolic processes that turn it over.^70^

To maintain homeostasis, cells need to couple the activities of TORC1 and PKA with the cell cycle. As we showed recently,^39^ this coupling results in fluctuations of the joint TORC1 and PKA activity in the cell cycle of WT mother cells. However, the extent to which the cell cycle machinery contributes to the generation of these fluctuations remains unclear. To assess this contribution, we used the OPTO-Cln2 system to alter the G1 duration and observe how TORC1/PKA activity dynamics adapt to the change. We therefore monitored the joint TORC1 and PKA activity in our OPTO-Cln2 *cln3Δ* strains under light and dark conditions, focusing on daughter cells where the greatest changes in G1 duration are observed between these conditions.

As a joint readout of TORC1 and PKA, we used the intracellular localization of Sfp1, a transcription factor that is targeted by both pathways.^39^^,^^71^ Active TORC1 and PKA phosphorylate Sfp1 and promote its accumulation in the nucleus, whereas inactivation of either pathway leads to a relocation of Sfp1 to the cytoplasm^71^^,^^72^^,^^73^ (Figure 4A). Monitoring the nuclear accumulation of GFP- and mNeonGreen-tagged Sfp1 during the cell cycle of individual WT mother cells had previously revealed two maxima, one in G1 and one in late G2, and we were able to associate the former with a peak in the joint TORC1 and PKA activity.^39^^,^^74^ GFP is not appropriate for monitoring signaling activity in our optogenetic strains, since its excitation spectrum overlaps with the absorption spectrum of EL222. We, therefore, switched to a red fluorescent protein and monitored the localization dynamics of mScarlet-I-tagged Sfp1 using time-lapse microscopy (Figure 4B). Consistent with our earlier observations,^39^ the addition of a fluorescent tag to Sfp1 produces a mild increase in cell doubling times (Figures S6A and S6B). The nuclear-to-cytosolic (N/C) ratio of Sfp1 was tracked during the cell cycle of both mother and daughter cells using a modification of our previously presented method to account for the lack of nuclear marker in our strains (STAR Methods, Figure S6C).

**Figure 4:**
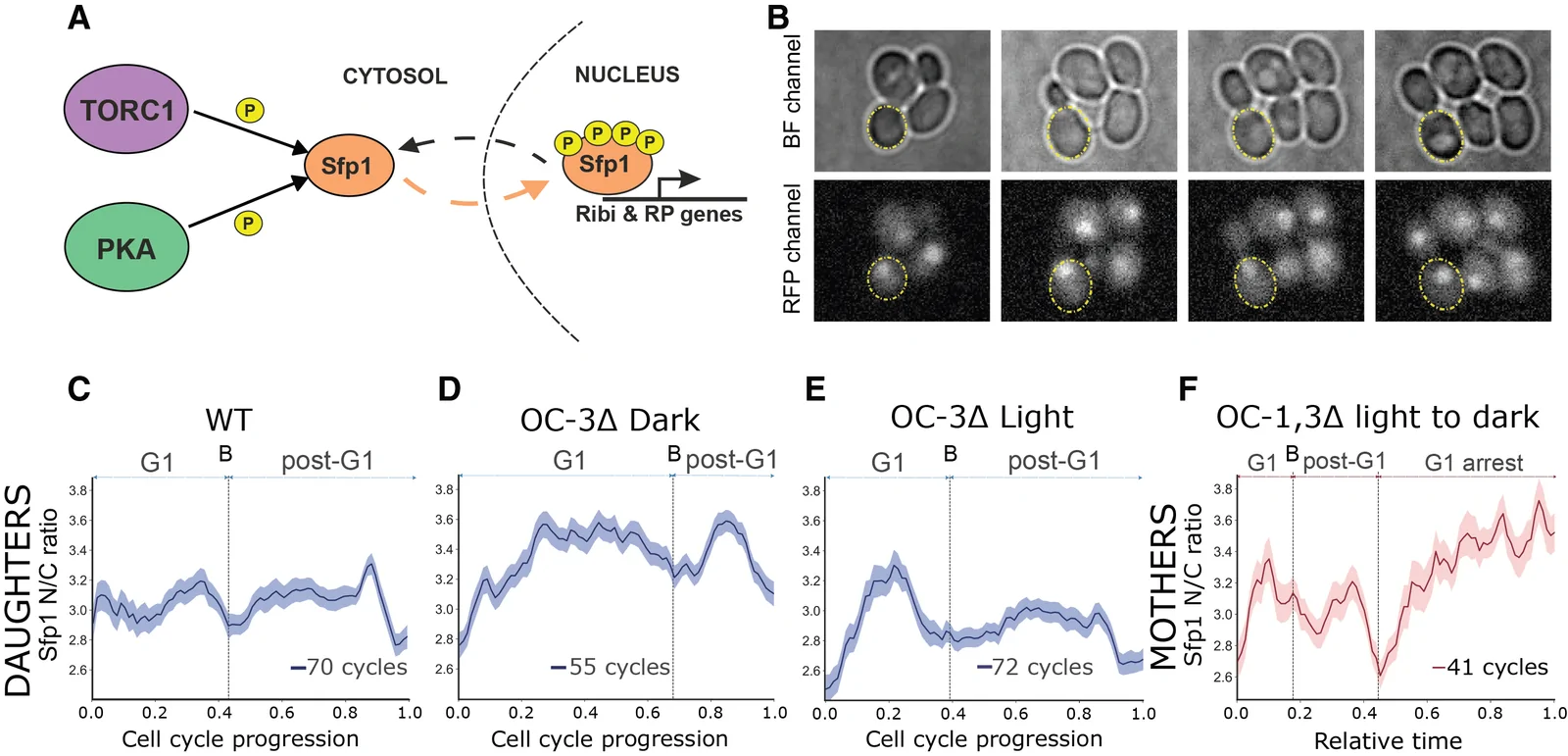
Combined TORC1 and PKA activity dynamics in response to light-induced changes in cell cycle progression (A) Schematic representation of the TORC1- and PKA-dependent regulation of Sfp1 phosphorylation and localization. (B) Representative bright-field and fluorescent microscopy images used to track Sfp1-mScarlet-I localization during the cell cycle. Cells were segmented (yellow ellipsoids), and the ratio of nuclear to cytosolic Sfp1 was calculated as indicated in STAR Methods. (C–E) Nuclear-to-cytosolic ratio of Sfp1 in aligned cell cycles of daughter cells. Cell cycle events (birth, budding [B], and cytokinesis) were manually annotated in microscopy movies. The length of G1 and post-G1 phases in each plot was based on the ratio of average G1 and post-G1 durations to the average cell cycle duration of each strain. The bands denote the 95% confidence interval for the mean. (F) Sfp1 nuclear to cytosolic ratio in light-grown OC 1,3Δ mother cells following arrest in the dark. The last cell cycle prior to arrest is shown along with the interval of G1 arrest. Single-cell trajectories were aligned at four points: the penultimate cytokinesis, the last budding (B), the last cytokinesis, and the last acquired time point (∼160 min after the end of the last completed cell cycle). The aligned trajectories were then averaged and plotted on a common (relative) time axis. The length of each interval in the plot is proportional to the average duration of each phase relative to the total experiment duration.

As previously reported,^39^ the localization of Sfp1-mScarlet-I exhibited an oscillatory pattern during the cell cycle of WT mothers, with maxima at G1 and G2 and minima around budding and cytokinesis (Figure S7A). This result confirms that mScarlet-I can be reliably used to monitor Sfp1 localization throughout the cell cycle, despite the absence of a nuclear marker, which increases the noise in individual time series. Similarly, mothers of the OPTO-Cln2 *cln3Δ* background displayed a G1 maximum under both under dark and light conditions (Figures S7B and S7C).

Sfp1 did not exhibit a clear localization pattern during the G1 of WT daughters (Figure 4C). Upon budding (i.e., entry into the cell cycle), the Sfp1 localization pattern of daughter cells became similar to that of mothers, with a maximum in G2 and a minimum around cytokinesis. Contrary to WT daughter cells, Sfp1 localization in dark-grown OPTO-Cln2 *cln3Δ* daughter cells displayed a substantial increase during G1 and a local minimum at budding (Figure 4D). Light-grown OPTO-Cln2 *cln3Δ* daughters showed a G1 localization peak that was similarly delimited by cytokinesis and budding (Figure 4E). Single-cell traces of Figures 4C–4E are presented in Figure S7E.

Collectively, these observations suggest that the combined TORC1 and PKA activity dynamics in OPTO-Cln2 *cln3Δ* daughters are mainly affected by changes in G1 progression. As G1 duration changes, the Sfp1 localization peak gets correspondingly adjusted, suggesting that the overall shape of TORC1 and PKA activity during G1 is mainly dictated by cell cycle-related processes.

We next asked how TORC1 and PKA respond when the cell cycle is halted. For this purpose, we tracked Sfp1 localization in OPTO-Cln2 *cln1Δcln3Δ* cells upon a transition from light to darkness. In this experiment, we followed mother cells that had budded shortly before or after the light was switched off and aligned their traces at their cytokinesis, the last budding event, the following cytokinesis, and the last time point at which each cell was tracked. Intriguingly, as the cells transitioned from their last cytokinesis into G1 arrest, the N/C ratio of Sfp1 increased considerably and was higher than the N/C ratio of the cell cycle preceding the arrest (Figure 4F). This increase cannot be attributed to Sfp1 accumulation after the arrest, since only the nuclear fraction of the protein increases (Figure S7F).

To determine how TORC1 and PKA contribute to the observed behavior of Sfp1, we monitored its localization following inhibition of either pathway. Rapamycin treatment of WT cells led to a rapid Sfp1 exit from the nucleus (Figure S7G, top left), while treatment with H-89, a kinase inhibitor with strongest effects on PKA,^75^^,^^76^^,^^77^ had a much smaller effect on Sfp1 localization (Figure S7G, top right). Interestingly, treatment of arrested cells with rapamycin did not delocalize Sfp1 from the nucleus (Figure S7G, bottom left). Similarly, treatment with H-89 did not result in Sfp1 delocalization (Figure S7G, bottom right). However, we observed that H-89 forms fluorescent aggregates and has relatively weak effects on WT cells (Figure S7G, top right), necessitating high concentrations to achieve partial inhibition. Because these high doses are delivered in DMSO, they induce growth defects that complicate the interpretation of Sfp1 localization changes.

Overall, our results indicate that Sfp1 becomes largely insensitive to the loss of TORC1 activity under arrest conditions. The role of PKA during arrest remains to be elucidated using more potent means of PKA inhibition, such as analog-sensitive PKA.^39^

### Light-driven arrest induces synchronous release of budding yeast cells

Besides offering the possibility to study the (de)coupling of growth and division mechanisms, the uniform and rapid arrest of OPTO-Cln2 *cln1Δcln3Δ* cells can be used to generate synchronous cell populations, which can, in turn, enable the study of cell cycle-dependent processes in liquid cultures using different omics methods. Contrary to traditional methods of cell cycle synchronization in which only a single round of arrest and release is typically possible due to technical limitations (e.g., inducer washout), using light in principle allows multiple rounds of arrest and release, and thus better temporal resolution of cell cycle processes away from the point of release.

To establish the optimal conditions for light-induced synchronization experiments, we first determined the percentage of cells that arrest after a transition of a light-proliferating culture to darkness. For this test, we sampled liquid cultures at different time points during a transition from light to darkness and calculated the percentage of budded vs. unbudded cells via microscopic inspection. Unbudded cells accounted for 89% of the population after 120 min of darkness and increased to 98% 180 min after the shift, demonstrating that our OPTO-Cln2 system can achieve a uniform cell-cycle arrest (Figure S8A).

As Figures 3H and S8A suggest, longer dark periods increase the percentage of arrested cells. On the other hand, prolonged arrest reduces the number of cells that can re-enter the cell cycle following a shift to light. Excessively long periods (>6 h) of arrest have adverse effects on cell morphology, as cells keep growing during the arrest and eventually die. To determine a dark period that optimizes the number of cells that can re-enter the cell cycle, we arrested cells grown in blue light under the microscope by shifting them to darkness for different lengths of time (80, 100, and 130 min) and calculated the percentage of budded cells upon release with another shift to blue light. We found that dark periods of 100 and 130 min both released ∼80% of the cells. On the other hand, we noticed that the 80-min arrest period was too close to the average cell cycle duration, which meant that a large fraction of cells had no time to reach G1 arrest before the light was switched on again. To reduce the effects of darkness on the cell morphology and enable multiple rounds of arrest and release, we proceeded with the optimized arrest period of 100 min.

To determine the minimal period of light induction that effectively releases the majority of cells for a single cycle before a second arrest, we performed experiments similar to the ones described above, with the periods of light and darkness reversed. These experiments established that 75 min of illumination is sufficient to release a large number of cells (∼90%) for a single cell cycle, while preventing entry into a second cell cycle (Figure S8B). Consequently, for repeated cycles of arrest and release, we used 100 min of darkness (arrest) followed by 75 min of light (release). Since culture conditions, media composition, and instrumentation may vary across laboratories, the optimal illumination and dark periods should be empirically determined for each experimental setup.

To benchmark the quality of synchronization achieved with our system, we compared its performance to the “gold-standard” arrest-and-release method for budding yeast, based on the alpha factor mating pheromone.^40^^,^^41^ Addition of the alpha factor to proliferating cells triggers a signaling cascade that ultimately leads to arrest in late G1^78^ (Figure 5A). Subsequent washout of the alpha factor allows cells to re-enter the cell cycle in a highly synchronous manner. For our tests, we arrested WT YSBN6 cells by alpha-factor (5 μg/mL) and OPTO-Cln2 *cln1Δcln3Δ* by a 100-min shift to darkness before their subsequent release (Figure 5A). We then used time-lapse microscopy to assess the release at the single-cell level, by monitoring the timing of bud appearance in the released cells. For the alpha-factor released cells, buds started to appear ∼55–80 min after washout, with the majority of budding events occurring ∼60 min post-washout (Figure 5B). For the OPTO-Cln2 *cln1Δcln3Δ* cells, we observed that the buds started appear between 35 and 85 min following light induction, with the majority of budding events occurring ∼55 min post-induction (Figure 5C). Overall, alpha factor and light induction displayed similar release dynamics.

**Figure 5:**
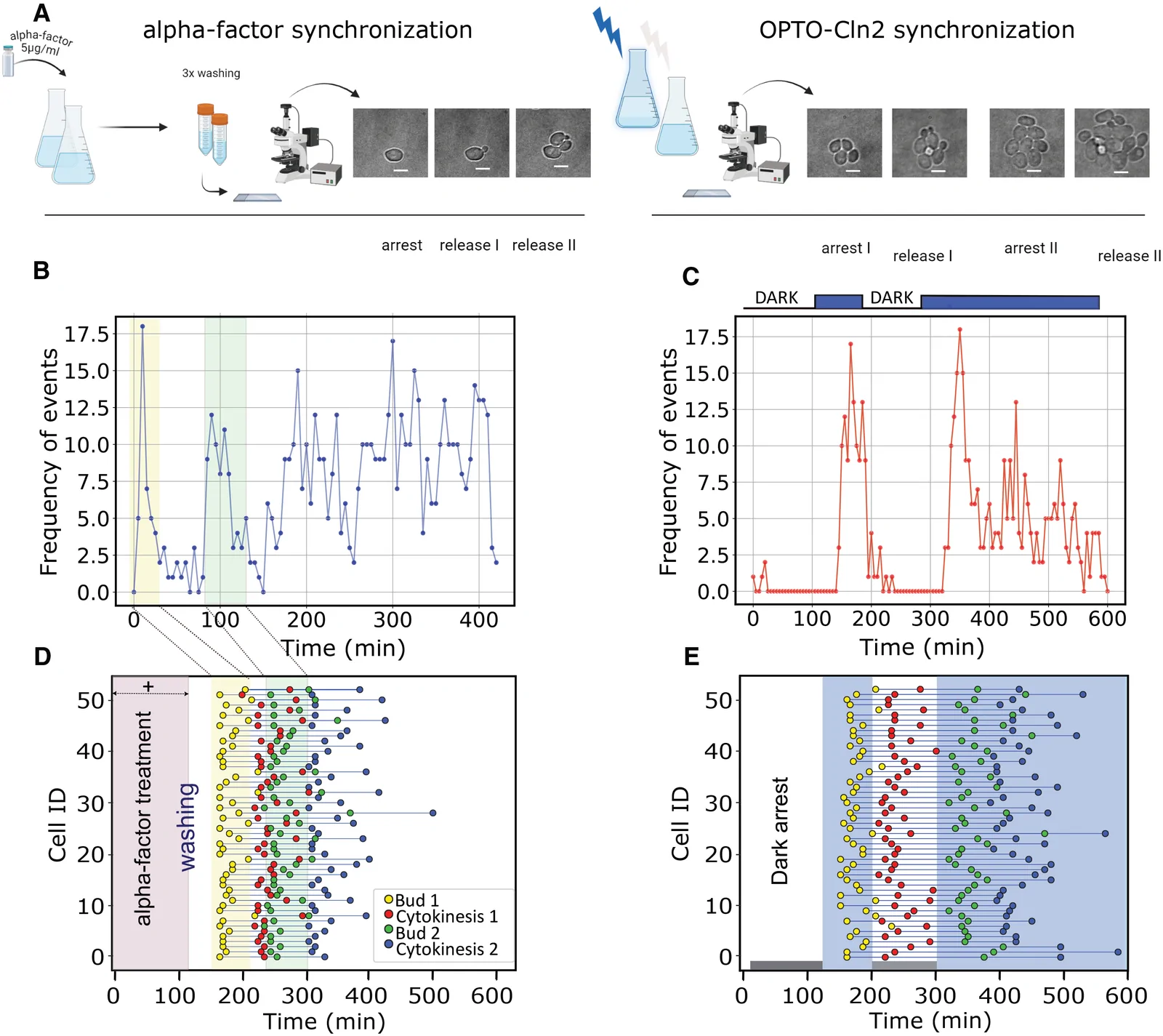
Comparison of alpha factor- and light-induced cell cycle synchronization (A) Schematic representation of experiments with alpha factor or light-induced synchronization. (B) Frequency of budding events following alpha factor arrest and release plotted against time. (C) Frequency of budding events following repeated light-induced arrest and release in OC-1,3Δ cells plotted against time. In total, 422 budding events were captured (251 events up to t = 400 min). (D) Cell cycle events of alpha factor-synchronized cells plotted against time, following two consecutive doublings after alpha factor release. (E) Cell cycle events of OC-1,3Δ cells during two consecutive light-induced arrests and releases plotted against time. It should be noted that the number of tracked cells in Figure 5E is lower than in Figure 5C.

To compare the degree of synchrony achieved by a single alpha factor release compared to two consecutive cycles of light-induced arrest and release, we determined the timing of cytokinesis and budding events of individual cells over time (Figure 5D). While budding events appear within a narrow time window following alpha factor washout, cytokinesis events of the first post-release cycle become intertwined with the budding events of the subsequent division cycle, making challenging the discrimination between the G2/M phase of the first cycle and the G1 phase of the subsequent cycle. On the other hand, repeated optogenetic arrest and release allows events from one division cycle to remain distinct from those of the subsequent cycle (Figure 5E).

Overall, our results demonstrate that the OPTO-Cln2 system can be used to induce population synchrony with quality comparable to that achieved by alpha factor, while avoiding the labor-intensive washout procedure required by the latter. Moreover, OPTO-Cln2 can induce repeated cycles of G1 arrest and release, providing better temporal resolution of late cell cycle events.

## Discussion

In this work, we employed a light-inducible system to dynamically perturb cell cycle progression in budding yeast through the regulation of the G1 cyclin Cln2. To benchmark our system, we characterized single and double G1 cyclin deletion mutants, focusing on cell volume and the duration of G1 and post-G1 phases. Although aspects of these phenotypes have been reported, a systematic analysis of the double mutant had been lacking. Consistent with prior studies,^50^^,^^55^^,^^79^^,^^80^^,^^81^ we observed an increase in cell size upon single cyclin deletion, with an even stronger effect in the double mutant.

Regarding cell cycle progression, both the *CLN3* and *CLN2,3* deletions induced a slight shortening of post-G1 phases, while deletion of *CLN2,3* strongly increased the length of G1 in daughter cells. The lack of a G1 delay in *cln3Δ*cells in both mothers and daughters has been reported previously,^7^ and so has the shortening of post-G1 phases. Here, we also find that *cln2Δcln3Δ* mothers share these features. Beyond providing benchmarks for validating the OPTO-Cln2 strains, these results are notable because they are not fully explained by current models of G1 regulation in budding yeast. Instead, they suggest compensatory mechanisms affecting the expression or timing of the remaining G1 cyclins, potentially linked to increased cell size. The connections between size control and cell cycle commitment remain to be elucidated. It is also important to note that G1 cyclin expression depends on the carbon source and deletion phenotypes differ between glucose (used here) and non-fermentable sources such as raffinose or glycerol.^82^^,^^83^ Thus, the generality of our observations across conditions remains to be determined.

Introduction of our OPTO-Cln2 construct in *cln3Δ* or *cln1Δcln3Δ* backgrounds allowed modulation of cell size and cell cycle progression consistent with expectations from the mutant characterization. Transitions between light- and darkness-associated phenotypes were rapid and uniform across cell populations. In this respect, OPTO-Cln2 performs comparably to the methionine-inducible Cln2 system^26^^,^^27^^,^^28^ and the estradiol-inducible Cln1 system,^29^^,^^30^^,^^31^ though the latter has not been extensively characterized. However, these systems require addition or removal of nutrients or hormones, making them best suited to microfluidic setups that allow rapid medium exchange. Moreover, nutrient removal and re-addition can affect nutrient-sensing pathways such as TORC1. In contrast, OPTO-Cln2 is compatible with both micro- and macroscopic (liquid culture) experiments because it uses light as an inducer. The high light sensitivity of the EL222^56^ enables activation with low intensities (<500 μW/cm^2^) that do not impair growth even over hours. The AQTrip EL222 variant used here further reduces light requirements, responding to brief (∼1 s) light pulses and remaining active for minutes. Under these conditions, the system behaves effectively as a binary switch rather than as a continuously tunable actuator. As a result, the choice of core promoter determines the maximal transcriptional output and basal leakage of the system, whereas light primarily controls the timing of activation.

An optogenetic cell cycle control system provides a useful tool for studying the coupling between the cell cycle machinery and growth-control processes. We found that the specific growth rate during G1 changed only modestly between WT, slowly dividing, and cell cycle-arrested daughters, suggesting that G1-associated volume growth can proceed largely independently of cell cycle progression. On the other hand, our tests with Sfp1, a joint TORC1 and PKA readout, suggest that the activity of these pathways is coordinated with cell cycle events at the G1 boundaries: in WT and optogenetic strains, mother cells show peak TORC1/PKA during G1 and minima at mitosis and budding. In *OPTO-Cln2 cln3Δ* daughters, a similar pattern is observed that scales with G1 duration under light and dark conditions Additional evidence comes from arrested *OPTO-Cln2 cln1Δcln3Δ* cells, where TORC1/PKA activity remains high, while cells continue to grow without re-entering the cycle. Our observation that Sfp1 localization becomes insensitive to TORC1 inhibition under these conditions resembles its behavior in cells lacking Bcy1, the regulatory subunit of PKA, which exhibit elevated PKA activity,^84^ suggesting that cell-cycle arrest may trigger PKA hyperactivation that overrides TORC1 control of Sfp1. Because H-89 is only a weak PKA inhibitor in yeast, this hypothesis would be better tested using analog-sensitive PKA alleles that allow more efficient inhibition.

Besides its utility in the study of coordination between growth and division, OPTO-Cln2 can serve as an arrest-and-release tool to induce population synchrony. In budding yeast, established synchronization methods include α-factor treatment, checkpoint-activating drugs, temperature-sensitive mutants, and centrifugal elutriation.^10^^,^^40^ Drug- and temperature-based approaches can induce stress responses and alter cell physiology, leaving α-factor and elutriation as the preferred methods. However, alpha factor rewires gene expression and signaling via the mating response,^85^^,^^86^^,^^87^ while elutriation yields relatively few early G1 cells and requires specialized equipment.^88^ Both approaches can also be technically demanding and variable. In contrast, light-based synchronization is simple and non-toxic and targets a single gene, minimizing off-target effects. It is also easily scalable to large liquid cultures, enabling omics-level analyses of transcription, translation, phosphorylation, and metabolism across the cell cycle. As with the other arrest-and-release strategies, cells continue to grow during arrest, which must be considered in experimental design. Although multiple cycles are possible, prolonged arrest can impair viability and complicate interpretation.

Given the good conservation of G1 progression mechanisms between yeast and mammalian cells,^1^ we expect that the core principle of our optogenetic system (the transcriptional control of G1 cyclins) can be extended to proliferating mammalian cells. More broadly, the ability to dynamically and reversibly control cell cycle progression provides a powerful framework for dissecting how growth and division are coordinated across eukaryotes.

### Limitations of the study

Long-term adaptation: OPTO-Cln2 is designed for short-term, acute perturbations of G1 progression. In cyclin deletion backgrounds maintained in darkness (i.e., without light-induced Cln2 expression), prolonged cultivation (72–96 h) may lead to progressive increases in growth rate, consistent with adaptive compensation. Likewise, OPTO-Cln2 *cln3Δ* and OPTO-Cln2 *cln1Δcln3Δ* kept under continuous induction over multiple days may also display altered growth rates, though we did not observe such effects when growing the cells for less than 48 h under constant blue light. These behaviors are not specific to OPTO-Cln2 but reflect a general feature of the systems involving conditional expression of cell cycle regulators in sensitized genetic backgrounds, where prolonged selective pressure can shift population dynamics.^36^^,^^89^ Accordingly, all experiments in this study were restricted to durations shorter than 48 h. For extended cultivation, growth rates and cell cycle phenotypes should be monitored to confirm stable system performance.

Quantification of Cln2 transcriptional dynamics: Cln2 transcripts turn over rapidly,^90^^,^^91^ making time-resolved RT-qPCR sensitive to delays during harvesting and processing. Moreover, increasing the temporal resolution of these experiments through frequent sampling can perturb culture conditions. Users aiming to resolve fast transcriptional transitions of OPTO-Cln2 should therefore optimize sampling procedures and balance temporal resolution against minimal disturbance of cell physiology.

## Resource availability

### Lead contact

Requests for further information and resources should be directed to and will be fulfilled by the lead contact, Andreas Milias-Argeitis (a.milias.argeitis@rug.nl).

### Materials availability

All plasmids and yeast strains generated in this study are available from the lead contact upon request.

### Data and code availability

• All data reported in this paper will be shared by the lead contact upon request.
• All original code has been deposited at Zenodo (https://doi.org/10.5281/zenodo.20643127) and is available in GitHub (https://github.com/amiliasargeitis/microscopy_scripts/tree/main/modified_scripts).
• Any additional information required to reanalyze the data reported in this work paper is available from the lead contact upon request.

## Acknowledgments

We would like to thank Prof. Mustafa Khammash (ETH Zurich, Switzerland) for providing the pDB146 plasmid and Michiel Punter (University of Groningen) for the cell cycle annotation ImageJ plugin. Figures 1A, 2A, 4A, and 5A were created using BioRender (BioRender.com). A.K. and A.M.-A. were supported by the Dutch Research Council (NWO) through an NWO-VIDI grant to A.M.-A. (project number 016.Vidi.189.116). C.L. was supported by the Dutch Research Council (NWO) through an NWO-M1 grant to A.M.-A. (project number OCENW.M.21.340).

## Author contributions

A.K. and A.M.-A. conceptualized and designed the study. A.K. generated the strains and performed and analyzed the experiments for the original submission. C.L. performed all experiments for the manuscript revision, with the exception of RT-qPCR, which was performed by D.I. A.K. and A.M.-A. wrote the manuscript. A.M.-A. acquired financial support and supervised the work.

## Declaration of interests

The authors declare no competing interests.

## STAR★Methods

### Key resources table

**Table:** 

REAGENT or RESOURCE	SOURCE	IDENTIFIER
**Bacterial and virus strains**		

Escherichia coli: DH5α	New England Biolabs	Cat#C2988J

**Chemicals, peptides, and recombinant proteins**		

Polyethylene glycol (PEG)	Sigma-Aldrich	Cat#202444
Carrier ssDNA (Deoxyribonucleic acid sodium salt from salmon testes)	Sigma-Aldrich	Cat#D1626
YPD Broth powder	Formedium	Cat#CCM0210
LB Broth Miller powder	Formedium	Cat#LMM0102
YNB w/o amino acids	Formedium	Cat#CYN0410
Complete Supplement Mixture (CSM)	Formedium	Cat#DCS0019
Yeast extract powder	Formedium	Cat#YEA02
Peptone	Formedium	Cat#PEP02
Glucose (D-(+)-Glucose monohydrate)	Sigma-Aldrich	Cat#49159
Agar	Formedium	Cat#AGR10
Bacto Tryptone	Thermo Fisher Scientific	Cat#211705
NaCl	Sigma-Aldrich	Cat#S9888
Lithium acetate dihydrate	Sigma-Aldrich	Cat#62393
Sodium dodecyl sulfate (SDS)	Sigma-Aldrich	Cat#L3771
Concanavalin A	Sigma-Aldrich	Cat# L7647
H-89	MedChemExpress	Cat# HY-15979A
Rapamycin	Sigma-Aldrich	Cat# R0395
TRIzol™	ThermoFisher Scientific	Cat#15596018
Chloramphenicol	Sigma-Aldrich	Cat#C0378
Ampicillin	Duchefa Biochemie	Cat#A0104
Kanamycin sulfate	Sigma-Aldrich	Cat#60615
G418 disulfate salt	Sigma-Aldrich	Cat#A1720
clonNAT	Jena Bioscience	Cat#AB-102
Hygromycin B	Duchefa Biochemie	Cat#H0192
T4 DNA Ligase Reaction Buffer	New England Biolabs	Cat#B0202S
T7 DNA Ligase	New England Biolabs	Cat#M0318S
BsmBI	New England Biolabs	Cat#R0580S
BsaI	New England Biolabs	Cat#R0535S
EcoRI-HF	New England Biolabs	Cat#R3101S
Q5 High-Fidelity DNA Polymerase	New England Biolabs	Cat#M0491S
5× Q5 reaction buffer	New England Biolabs	Cat#B9027S
10× Buffer rCutSmart	New England Biolabs	Cat#B6004S
PvuII-HF	New England Biolabs	Cat#R3151S

**Critical commercial assays**		

Miniprep kit	MACHEREY-NAGEL	Cat#740588
PCR purification kit	MACHEREY-NAGEL	Cat#740609

**Experimental models: Organisms/strains**		

*Saccharomyces cerevisiae* YSBN6 (MATa HIS3 LEU2 MET15 URA3 ho:HphMX4)	Canelas et al.^42^	N/A
yAK29.2.3α (YSBN6 *Whi5:WHI5-mCherrry:Ble, ho::ACT1p-VP-EL222-CYC1t-hphMX4*)	This study	N/A
yAK31.1.1α (YSBN6 *ho::ACT1p-VP-EL222-CYC1t-hphMX4, cln2:cln2Δ, cln3:cln3Δ*)	This study	N/A
yAK65.2.2α (YSBN6 *ho::prACT1-VP-EL222-CYC1-hphMX4, cln2::El222(5xBS)-prSPO13-CLN2-mNG, cln3:cln3Δ*)	This study	N/A
yAK35.1.3α (YSBN6 *Whi5:WHI5-mCherrry:Ble, ho::prACT1-VP-EL222-tCYC1-hphMX4, cln3:cln3Δ*)	This study	N/A
yAK61.1.5α (YSBN6 *WHI5-mCherry:Ble, ho::prACT1-VP-EL222-CYC1-hphMX4, El222(5xBS)-prSPO13-CLN2-mNG, cln3:cln3Δ, cln1:cln1Δ*)	This study	N/A
yAK71.2.3γ (YSBN6 Whi5:*WHI5-mCherry:Ble, ho::prACT1-VP-EL222-CYC1-hphMX4, cln2:El222(5xBS)-prGAL1-CLN2-mNG, cln3:cln3Δ*)	This study	N/A
yAK76.1.4 (YSBN6 *ho::HphMX4, Sfp1:SFP1-mScarlet-I-NatMX*)	This study	N/A
yAK78.1 (YSBN6 *ho::prACT1-VP-EL222-CYC1-hphMX4, cln2:El222(5xBS)-prSPO13-CLN2-mNG, cln3:cln3Δ, Sfp1:SFP1-mScarlet-I-NatMX*)	This study	N/A
yAK78.2 (YSBN6 *ho::prACT1-VP-EL222-CYC1-hphMX4, cln2:El222(5xBS)-prSPO13-CLN2-mNG, cln3:cln3Δ, cln1:cln1Δ, Sfp1:SFP1-mScarlet-I-NatMX*)	This study	N/A
ySC3.1.1α (YSBN6 Whi5:*WHI5-mCherry:Ble, ho::prACT1-VP-EL222-CYC1-hphMX4, X1::El222(5xBS)-prSPO13-CLN2-mNG, cln3:cln3Δ*)	This study	N/A
RF1.1 (YSBN6 Whi5:*WHI5-mCherry:Ble, ho::prACT1-VP-EL222-CYC1-hphMX4, cln2::El222(5xBS)-prSPO13-CLN2-mNG (1000bp truncation), cln3:cln3Δ*)	This study	N/A

**Oligonucleotides**		

Primers	This paper	see Supplemental Table S2A
CRISPR oligos	This paper	see Supplemental Table S2B

**Recombinant DNA**		

pYTK-DN1 (selection: ampicillin)	Novarina et al.^92^	Addgene Plasmid #180282
pYTK-DN2 (selection: ampicillin)	Novarina et al.^92^	Addgene Plasmid #180283
pYTK-DN5 (selection: kanamycin)	Novarina et al.^92^	Addgene Plasmid #180286
pAK145: EL222(5xBS)-pSPO13-Kozak-CLN2-mNG	This study	N/A
pSC1.1.3: pFA6-GAL(UAS)-EL222(5xBS)-pGAL1-CLN2-mNG	This study	N/A
pLV1: ACT1pr -VPEL222(AQTrip)-CYC1term	Guerra et al.^93^	N/A
pYTKA123: Cas9 + HO-sgRNA	This study	N/A
pYTAK140: Cas9 + Cln2(TU2)/Cln3(TU3)	This study	N/A
pDB146: ACT1pr-mScarlet-I-VPEL222(AQTrip)-CYC1term	Rullan et al.^68^	N/A
pSfp1_mNG_Nat: pFA6-SFP1-mNeonGreen-NatMX	Guerra et al.^39^	N/A
pSfp1-mScarlet-I: pFA6-SFP1-mScarlet-I-NatMX	This study	N/A
pYTAK146: Cas9 + X1-sgRNA	This study	N/A
pGG3_Whi5: Cas9 + WHI5-sgRNA	Chen et al.^94^	N/A

**Software and algorithms**		

E-CRISP	Heigwer et al.^95^	http://www.e-crisp.org/
SnapGene Viewer	SnapGene	https://www.snapgene.com/snapgene-viewer/
Benchling	Benchling	https://benchling.com/editor
Fiji	Schindelin et al.^96^	https://imagej.net/software/fiji/downloads
RStudio (R version 4.5.0)	RStudio	https://www.rstudio.com/
Python (version 3.7)	Python Software Foundation	https://www.python.org
Custom analysis scripts (Python, ImageJ Macro)	This study	https://doi.org/10.5281/zenodo.20643127

**Other**		

T100 Thermal Cycler	Bio-Rad	Cat#186-1096
Nikon Eclipse Ti-E	Nikon Instruments	
LED Uni-Color Blue 470nm	Lumex Opto/Components Inc.	SSL-LX5093USBC
μ-Slide 8 Well	ibidi	Cat# 80821
Thermo Savant Fastprep FP120	Thermo Scientific	FP120-230

### Experimental model and study participant details

#### Yeast strains and culture conditions

All strains used in the study are derivatives of the S288C-derived prototrophic genetic background YSBN6^42^ (Euroscarf, Germany). For all experiments, yeast cells were grown in YNB medium (Formedium) supplemented with amino acids and 2% glucose. All liquid cultures were cultivated at 30°C with shaking at 300 rpm. To ensure exponential growth at the start of our experiments, precultures were appropriately diluted in the evening prior to each experiment, so that cells would be in mid-log phase (OD < 1) on the day of the experiment. For experiments starting from dark conditions, precultures were grown in flasks covered with aluminum foil, and cell sampling was conducted in a dark room illuminated with a red LED light. For light-grown precultures, illumination was provided by a breadboard carrying 3 or 4 blue LEDs (Lumex, part no. SSL-LX5093USBC, 470 nm) that was attached to the incubator wall facing the preculture flask (Figure S8C).

### Method details

#### Plasmid and strain construction

CRISPR plasmids targeting the HO, *WHI5*, *CLN1*, *CLN2* and *CLN3* loci were prepared using a previously described protocol.^92^ For the construction of each plasmid, we used pYTK-DN1 (containing the Cas9 cassette), pYTK-DN2 (spacer cassette), a double-stranded sgRNA and pYTK-DN5 (antibiotic resistance marker) from ref.^92^ Individual sgRNAs were selected using the E-CRISPR tool following the guidelines of ref.^92^ to ensure specificity. In the case of *CLN2*, the sgRNA was targeted at the promoter sequence to avoid modifying the coding sequence with synonymous mutations.

For the construction of the EL222 repair fragment (introduced into the HO locus), we modified plasmid pDB146^68^ (kindly donated by Prof. Mustafa Khammash, ETH Zurich) by removing mScarlet-I, leaving *ACT1*pr-VPEL222_AQTrip-CYC1t. For the construction of the repair fragment containing an EL222-sensitive promoter upstream of the *CLN2* ORF, Gibson assembly was used to introduce EL222 binding sites (5×), the SPO13 core promoter,^56^ a Kozak sequence,^56^ the *CLN2* ORF and an mNeonGreen tag in a pFA6 backbone (mNeonGreen fluorescence of this construct was not clearly distinguishable from cellular autofluorescence and was therefore not used in this study). For the construction of Sfp1-mScarlet-I, mScarlet-I was PCR amplified from pDB146 and was used to construct pFA6-SFP1-mScarletI-NATMX via Gibson assembly. Since OPTO-Cln2 and cyclin deletion strains carry a Whi5-mCherry fusion, CRISPR was used to remove mCherry prior to Sfp1-mScarlet-I genomic integration. The plasmids containing the repair fragments were used as PCR templates, where long primers with tails homologous to the corresponding genomic integration locus were used to amplify the region of interest. All CRISPR target sequences and repair fragment tails used for genomic integration are listed in Table S2. All genomically integrated constructs were verified by PCR and Sanger sequencing (Eurofins genomics, the Netherlands).

#### Growth curve analysis

Yeast cells were cultured as described above. To ensure steady-state growth, liquid cultures were maintained in exponential phase (OD_600_ < 1) for at least 24 h prior to the experiment. The OPTO-Cln2, *cln3Δ* strain was pre-cultured in darkness and exposed to light only on the day of the experiment for the OC(L) condition. Samples were collected hourly, and cell concentrations were determined via flow cytometry, in a BD Accuri C6 cytometer. Three independent biological replicates were performed for each strain, with measurements obtained on separate days to ensure reproducibility.

#### Microscopy

Microscopy experiments were performed using inverted fluorescence microscopes (Eclipse Ti-E, Nikon Instruments). Temperature was kept at 30°C with the help of a temperature-controlled microscope incubator (Life Imaging Services). All images were obtained with a 100× Nikon S Fluor (N.A. = 1.30) objective and were recorded using iXon Ultra 897 DU-897-U-CD0-#EX cameras (Andor Technology). For fluorescent microscopy, LED-based excitation systems (pE2; CoolLED Limited and AURA; Lumencor) were used. For red fluorescent protein (mScarlet-I) measurements, cells were excited at 565 nm (excitation filter: 540–580 nm, dichroic: 590 nm, and emission filter: 600–650 nm). To activate the EL222 system through the microscope, we used a LED light source (CoolLED pE2) centered at 440 nm at 1% intensity, illuminating the cells for 1 s every 5 min. During brightfield imaging, a long-pass (600 nm) filter was used to prevent unwanted activation of the EL222 system. The Nikon perfect focus system was used to prevent loss of focus in all experiments, and image acquisition was performed every 5 min.

For the determination of cell cycle phase durations and cell volumes, mid-log yeast cells grown on YNB supplemented with amino acids and 2% glucose were spotted on a microscopy slide and covered with an agarose pad (YNB +aa +2% glycose +1% agarose). Whenever necessary, sample preparation for microscopy was performed in a dark room using a red-light LED lamp to avoid sample exposure to ambient blue light. Prior to each experiment, the samples were kept at the microscope for ∼1−2 h for cells to adapt to the new growth environment. For experiments requiring light-grown precultures, cells that were transferred to the microscope were kept illuminated by an LED breadboard (described above) during the acclimation period (Figure S8C).

The experiments involving chemical perturbations with rapamycin and H89 were carried out in plastic chambered coverslips (μ-Slide 8 Well, Ibidi). The coverslips were coated with concanavalin A (1 mg/mL; Sigma-Aldrich) by loading 250 μL of concanavalin in each well and incubating for 10 min in a sterile environment. Subsequently, the wells were washed with 250μL sterile ddH2O and were dried for 20 min. 300μL of exponentially growing cells were added to the wells at an OD600 of 0.1 and were incubated for 30 min at 30°C. The wells were then washed with pre-warmed medium twice to remove unattached cells. The attached cells were imaged under the microscope in the brightfield and RFP channels using the imaging settings described above. The light-sensitive strain (OPTO-Cln2 *cln1Δcln3Δ*) was transferred to darkness 2.5 h before drug addition. Cells were treated with the following chemicals: the drug vehicle DMSO (2% final concentration), rapamycin (Sigma-Aldrich, final concentration 200 ng/mL, diluted in DMSO), and H-89 (MedChemExpress, final concentration 100μM, diluted in DMSO).

#### Generation of Sfp1–mScarlet-I nuclear-to-cytosolic ratio plots

To compare and average the Sfp1 N/C ratio during cell cycles of different lengths, individual cell cycle traces were aligned at three cell cycle events: cytokinesis (birth for daughters), budding (end of G1), and the subsequent cytokinesis event. The traces were interpolated with the same number of time points on an axis representing cell cycle progression, by allocating to each phase (G1 and post-G1) a number of points proportional to the average duration of that phase in a given population. The aligned and interpolated traces were subsequently averaged to obtain the localization patterns presented here.

#### RNA extraction

Liquid cultures were grown as described above and maintained in exponential phase (OD_600_ < 1) for at least 24 h prior to pellet collection. For dark/light mRNA level comparisons (Figure S4A), the OPTO-Cln2 *cln3Δ* strain was kept in darkness during all pre-culturing steps. After collecting the dark sample, the same culture was exposed to light for 2 h before the collection of the light sample. OPTO-Cln2 *cln1Δcln3Δ* was grown under low-intensity blue light illumination using the setup shown in Figure S8C. Following the collection of the light sample, the same culture was shifted to darkness for 2 h before collection of the dark sample. For light transition experiments (Figure S4B), separate cultures were prepared under the same pre-culturing conditions and light/dark shift directions, but cells were harvested 30 min after transfer to the new light condition.

From each culture, a volume containing 3 × 10^7^ cells (determined by cell counting) was collected and centrifuged at 2,300g for 3 min at 4°C. Cell pellets were snap-frozen in liquid nitrogen, then resuspended in 500 μl ice-cold TRIzol Reagent (cat. 15596018, ThermoFisher Scientific). 0.25 g glass beads were then added and samples were disrupted in a Savant Fastprep FP120 beadbeater (Thermo Scientific) for 1 min. Samples were then incubated on ice for 1 min, after which beating was repeated once. An additional 500 μl TRIzol Reagent were then added and samples were incubated at 70°C for 20 min, before proceeding with RNA extraction as per manufacturer instructions.

#### RT-qPCR

RT-qPCR was performed using the Luna Universal One-Step RT-qPCR Kit (cat. E3005X, New England Biolabs), as per manufacturer instructions. A relative standard curve was prepared by mixing all samples and by preparing serial dilutions of this pool at 200-100-50-25-12.5-6.25 ng/ul. For sample quantification, each sample was diluted to achieve a concentration of 50 ng/ul and 1 μl was used for each RT PCR reaction. The following primers were used: ACT1 (For: CGTCGGTAGACCAAGACACC, Rev: GTGTTCTTCTGGGGCAACT), UBC6 (For: AAGGGCGGTCAATATCACGG; Rev: TCCTGTCGTGGCTTCATCAC), and CLN2 (For: TGGTCAGCAGCAGTCAAACA; Rev: CCGTTGCGGGATTTTGGTTT). Sample relative concentrations were determined by interpolating to the standard curve. CLN2 concentration was normalized over ACT1 or UBC6.^97^

#### Light-induced synchronization (with cell fixation)

Log-phase (OD < 1) light-grown cultures were covered with aluminum foil to induce cell cycle arrest and were kept in a 30-C incubator. Sample collection was performed every 30 min by pelleting 1 ml of culture, resuspending in ice-cold 4% formaldehyde fixation solution, and washing in ice-cold PBS. Individual time point samples were stored at 4°C prior to microscopic quantification. For this quantification, 2 μl of each sample were spread on a microscopy slide and covered with an agar pad infused with 1× PBS solution and 1% agarose. Acquisition of multiple fields per sample was performed using the custom multipoint definition in NIS Elements, set to scan an area of 10 × 10 fields using 1% overlap, which resulted in 100 images for each sample. The percentage of budded vs. unbudded cells in each image was then calculated by visual inspection.

#### Alpha-factor treatment

Before the addition of alpha factor, precultures of exponentially growing WT cells were diluted to OD 0.2 in 20ml YNB supplemented with amino acids and 2% glucose. A stock of 0.5 mg/ml alpha-factor (Eurogentec, Belgium) was diluted to a working concentration of 5 μg/ml before the addition in the culture. After alpha factor addition, the cultures were left at 30°C for 2h. Subsequently, the alpha factor was washed to release the cells into the cell cycle. For alpha factor wash-off, cells were centrifuged (2000 rpm for 2 min) and resuspended 3 times in 2× volume of YNB medium without alpha-factor before being resuspended in 1× volume of YNB. For fixation experiments, samples were collected every 30 min as described above. For the microscopy experiments with alpha factor treatment, cells were kept in the incubator without disturbance for the duration of the treatment (2h). Following alpha factor washout (time zero in Figure 5C), microscopy experiments were performed in YNB-agar pads as indicated above.

### Quantification and statistical analysis

#### Statistical tests

All statistical comparisons were performed using non-parametric Mann-Whitney U tests and effect sizes were calculated using the rank-biserial correlation. Sample sizes, exact *p*-values, and comparison-specific details are provided in the corresponding captions of figures and Supplementary figures. For descriptive analyses, details are indicated in the corresponding captions of figures and Supplementary figures.

#### Generation of distribution plots

Histograms and violin plots were generated in Python using the Seaborn library. For all histograms of cell cycle phase durations, a bin size of 5 min was used. The y axis values represent normalized bin frequencies and therefore have no physical units. Kernel density estimation (KDE) curves were computed using the seaborn.kdeplot() function (default settings) and overlaid on the histograms as visual aids. Violin plots were generated using the seaborn.violinplot() function (default settings), which applies KDE-based smoothing to represent the underlying data distribution.

#### Image analysis

All brightfield images were contrast-enhanced to facilitate segmentation, and fluorescent images were background corrected using the rolling ball subtraction plugin of ImageJ. For cell segmentation and tracking in time-lapse experiments, brightfield images were processed with the semi-automatic ImageJ plugin BudJ.^98^ BudJ was used to provide estimates of cell volumes, as well as segmentation masks for processing the fluorescent channel images. Analysis of the BudJ-generated data and fluorescent images was performed using a custom-made script Python script.

Due to the fact that the absorption spectrum of EL222 extends up to 500 nm^57^ and Sfp1 was tagged with a red fluorescent protein (mScarlet-I), we could not use a nuclear marker as in our previous work^39^ to accurately locate the yeast nucleus. We therefore used a modified procedure for the estimation of nuclear and cytoplasmic fluorescence, in which the fluorescence intensity of Sfp1 itself (which is always partially nuclear in rich nutrients) was used to define an area of brightest pixels via local thresholding (threshold_local() from the scikit-image Python library) within each cell mask. By varying the offset parameter of the local threshold for each cell, we determined a bright region that contains no more than 30% of the total cell pixels, a percentage that is slightly larger than the average fraction of nuclear to whole-cell area.^99^ The resulting region was then eroded using a 3 × 3 square structuring element to avoid inclusion of small non-connected regions of bright pixels, leaving only a region of contiguous pixels that should be located within the nucleus (nuclear ROI). The nuclear intensity of Sfp1 was then estimated from the average fluorescence of a circular region at the center of the nuclear ROI with a radius of three pixels. To estimate the cytosolic signal, a larger concentric circle with a radius of nine pixels was defined to ensure exclusion of nuclear pixels, and the average fluorescence of pixels between the cell boundary and the boundary of this larger circle was calculated (Figure S6C). The average nuclear and cytosolic fluorescence were in turn used to calculate the nuclear-to-cytosolic ratio of Sfp1.

#### Determination of cell cycle phase durations

To estimate the doubling times and (post-)G1 durations of individual cells, a custom-made ImageJ plugin (Click_cells.ijm, available at https://doi.org/10.5281/zenodo.20643128) was used to manually annotate events (birth, budding, cytokinesis and death) for tens of cells throughout a microscope video. The output of the ImageJ plugin was further analyzed using a Python script to calculate standard statistics and make plots. The annotation of individual events was based on visual inspection of cellular features. Birth of daughter cells (and cytokinesis) was assigned to the time point at which a dark segment was visible between the mother and daughter cell, implying that cytokinesis had been completed. Budding was assigned to the time point at which a dark spot, indicating the nascent bud, was visible on the periphery of a cell. The G1 duration of a daughter cell was then defined as the length of the interval between its birth and first budding event. Likewise, the G1 duration of a mother cell was defined as the length of the interval between a cytokinesis event and the following budding event. The doubling time of a mother cell was calculated as the length of the interval between two consecutive budding events. Finally, the S/G2/M (or post-G1) duration of a cell was defined as the length of the interval between budding and the following cytokinesis event. Note that, due to the use of a 5-min sampling interval (cf. Microscopy section), the medians of all durations reported in the main text are multiples of 5.

## Supplemental information

Document S1. Figures S1–S8

Table S1. Statistics of cell cycle phase durations, cell volumes at budding, and volume increase rates for the strains analyzed in this study, related to Figures 1–3

Table S2. Primers and CRISPR-related oligos used in this study, related to the key resources table

Document S2. Article plus supplemental information
