# Supplementary material for: Rapid and reversible regulation of cell cycle progression in budding yeast using optogenetics
Source: Cell Rep Methods. 2026 Jul 30;6(10):101541. doi: 10.1016/j.crmeth.2026.101541 (PMC13721752; doi:10.1016/j.crmeth.2026.101541)
Supplement: Supplementary file 1 [file mmc1.pdf]

Cell Reports Methods, Volume *6*

Supplemental information

Rapid and reversible regulation of cell cycle progression in budding yeast using optogenetics

Andriana Koutsoumpa, Catherine Loukogiannaki, Danny Incarnato, and Andreas Milias-Argeitis

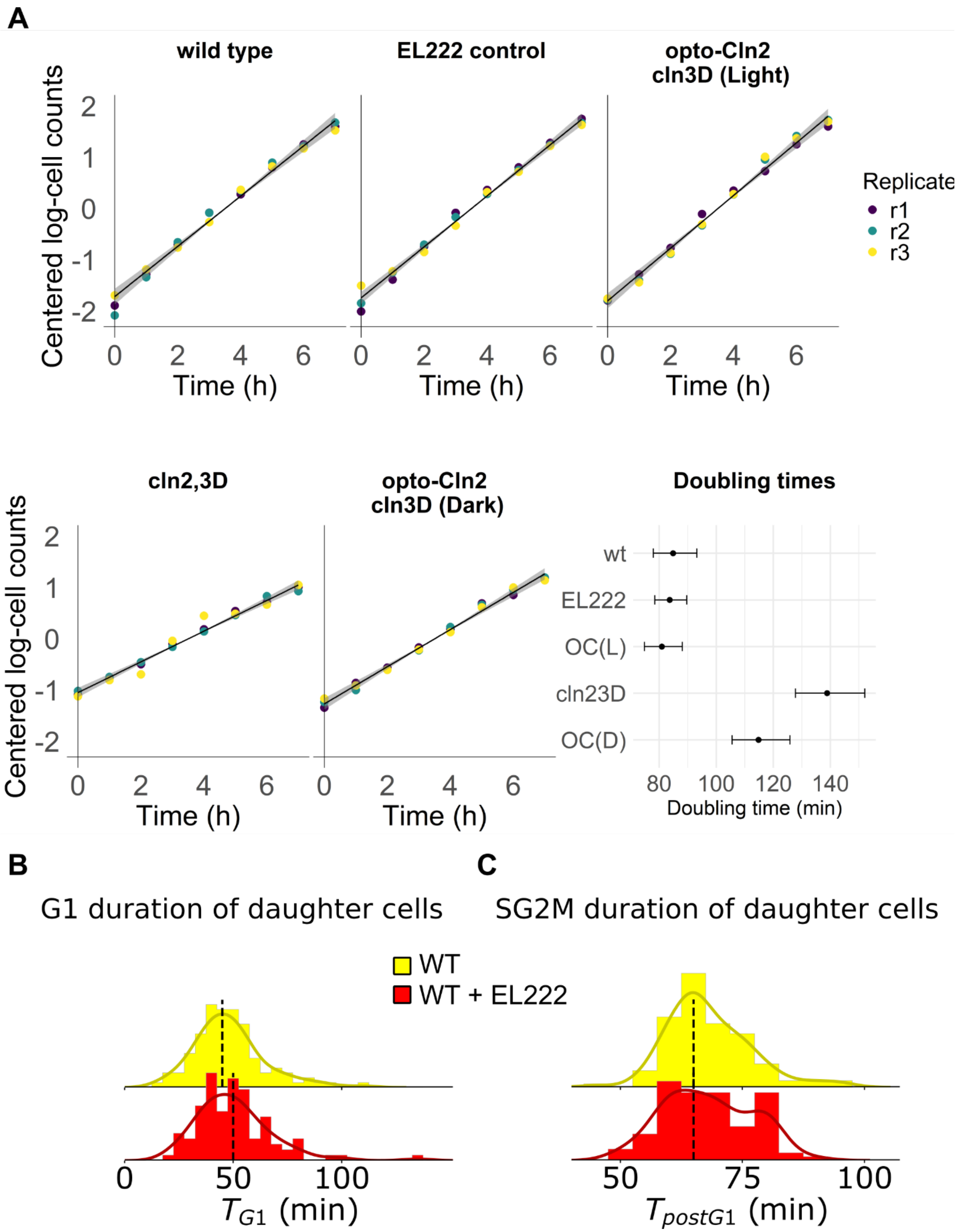

A
wild type
EL222 control
opto-Cln2
cln3D (Light)
Centered log-cell counts
Time (h)
Replicate
r1
r2
r3
cln2,3D
opto-Cln2
cln3D (Dark)
Doubling times
wt
EL222
OC(L)
cln23D
OC(D)
80
100
120
140
Doubling time (min)
B
G1 duration of daughter cells
C
SG2M duration of daughter cells
WT
WT + EL222
T_{G1} (min)
T_{postG1} (min)

Figure S1. Proliferation dynamics of wild-type, cyclin mutants and OPTO-Cln2 strains. Related to Figures 1 and 2. **A)** Growth curves of WT, WT with EL222 AQ-Trip integrated at the HO locus, cln2Δcln3Δ (with EL222 AQ Trip at the HO locus) and OPTO-Cln2 strains grown in YNB medium supplemented with amino acids and 2% glucose (STAR Methods). OPTO-Cln2 liquid pre-cultures were grown in the corresponding light or darkness conditions prior to each experiment, and all strains were maintained in the exponential phase (OD600 < 1) for at least 24 h prior to measurement acquisition. The plots display three independent experiments per strain, each performed on a different day. To visualize the data on a common logarithmic scale, the average of the log-transformed cell counts from each experiment was subtracted from all corresponding data points, centering each experiment's log-counts at zero. The normalized data points of each experiment are displayed as dots in different colors. Growth rates and their associated standard errors were obtained by linear regression of log-counts versus time for each individual experiment. The three independent growth rate estimates were then combined using the rma() function of the metafor R package (method = "FE"), which calculates a weighted average of effect sizes under a fixed-effects model, using the inverse of their variances as weights. The overall growth rate and its standard error were used to calculate 95% confidence intervals. These were subsequently transformed into the doubling time confidence intervals shown in the plots, using the relation $\tau = \ln(2)/\mu$, where μ is the growth rate and τ is doubling time of cell counts. The fitted line in each plot corresponds to the overall growth rate estimate, and the shaded region represents its associated 95% prediction band. **B)** and **C)** Distributions of G1 and post-G1 durations of WT daughters and daughters expressing EL222 construct at the HO locus. Statistical comparisons, two-tailed Mann-Whitney test: WTG1 – EL222G1, $p = 0.4$, $|r| = 0.07$, WTpost-G1 – EL222post-G1, $p = 0.9$, $|r| = 0.09$.

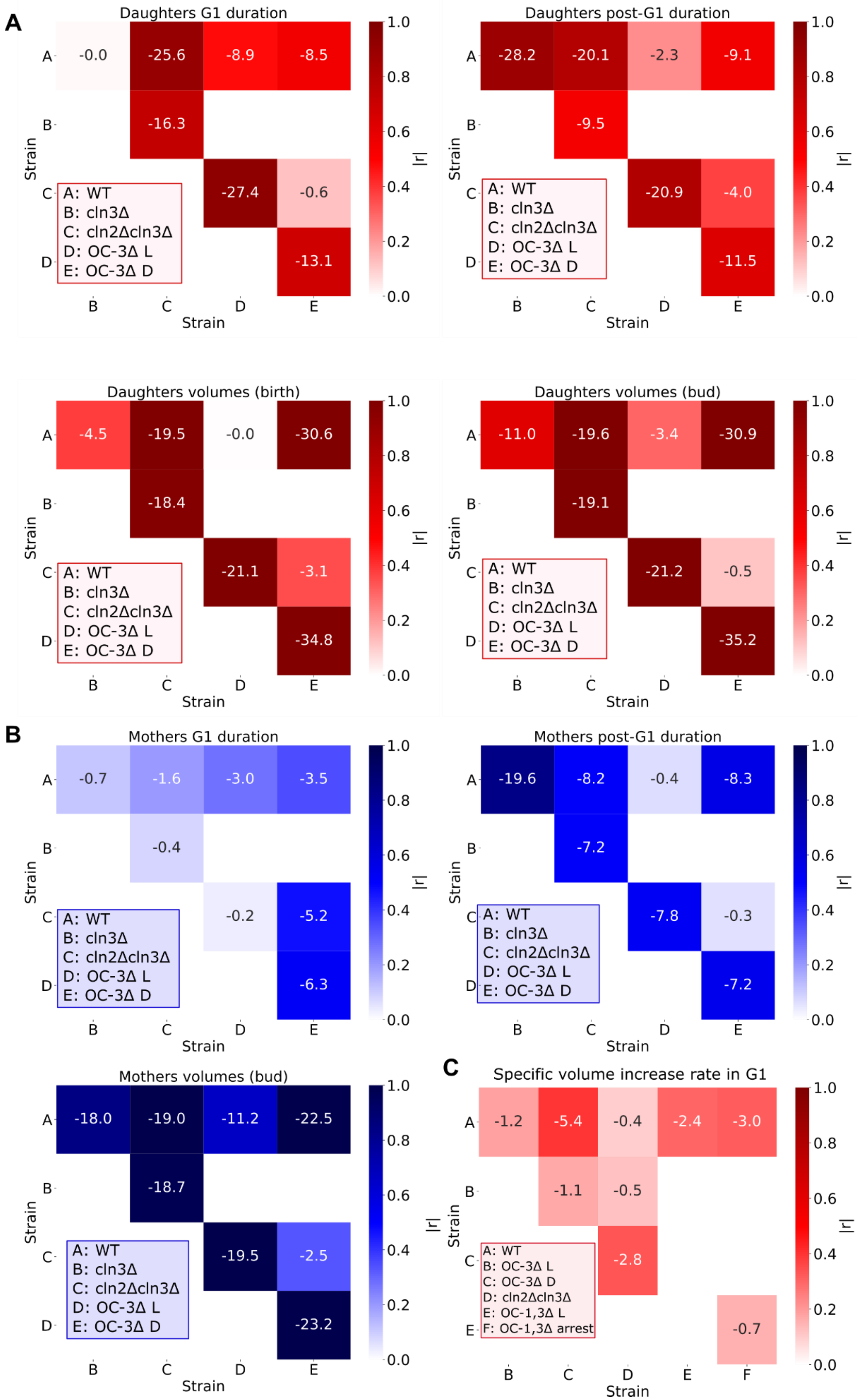

A
Daughters G1 duration
Daughters post-G1 duration
Daughters volumes (birth)
Daughters volumes (bud)
B
Mothers G1 duration
Mothers post-G1 duration
Mothers volumes (bud)
C
Specific volume increase rate in G1
Strain
|r|
A: WT
B: cln3Δ
C: cln2Δcln3Δ
D: OC-3Δ L
E: OC-3Δ D
A: WT
B: OC-3Δ L
C: OC-3Δ D
D: cln2Δcln3Δ
E: OC-1,3Δ L
F: OC-1,3Δ arrest

Figure S2. Graphical summary of statistical comparisons of G1 and post-G1 durations, cell volumes at birth and budding, as well as specific growth rates for cyclin mutants and OPTO-Cln2 strains. Related to Figures 1, 2 and 3. **A)** Comparisons of daughter G1/ post-G1 durations and cell volumes **B)** Comparisons mother G1/ post-G1 durations and cell volumes, **C)** Comparisons of daughter specific volume increase rates during G1. In each matrix, numbers represent the Mann-Whitney test $\log_{10}$(p-value), and color intensity corresponds to effect size (absolute value of rank-biserial correlation (r)).

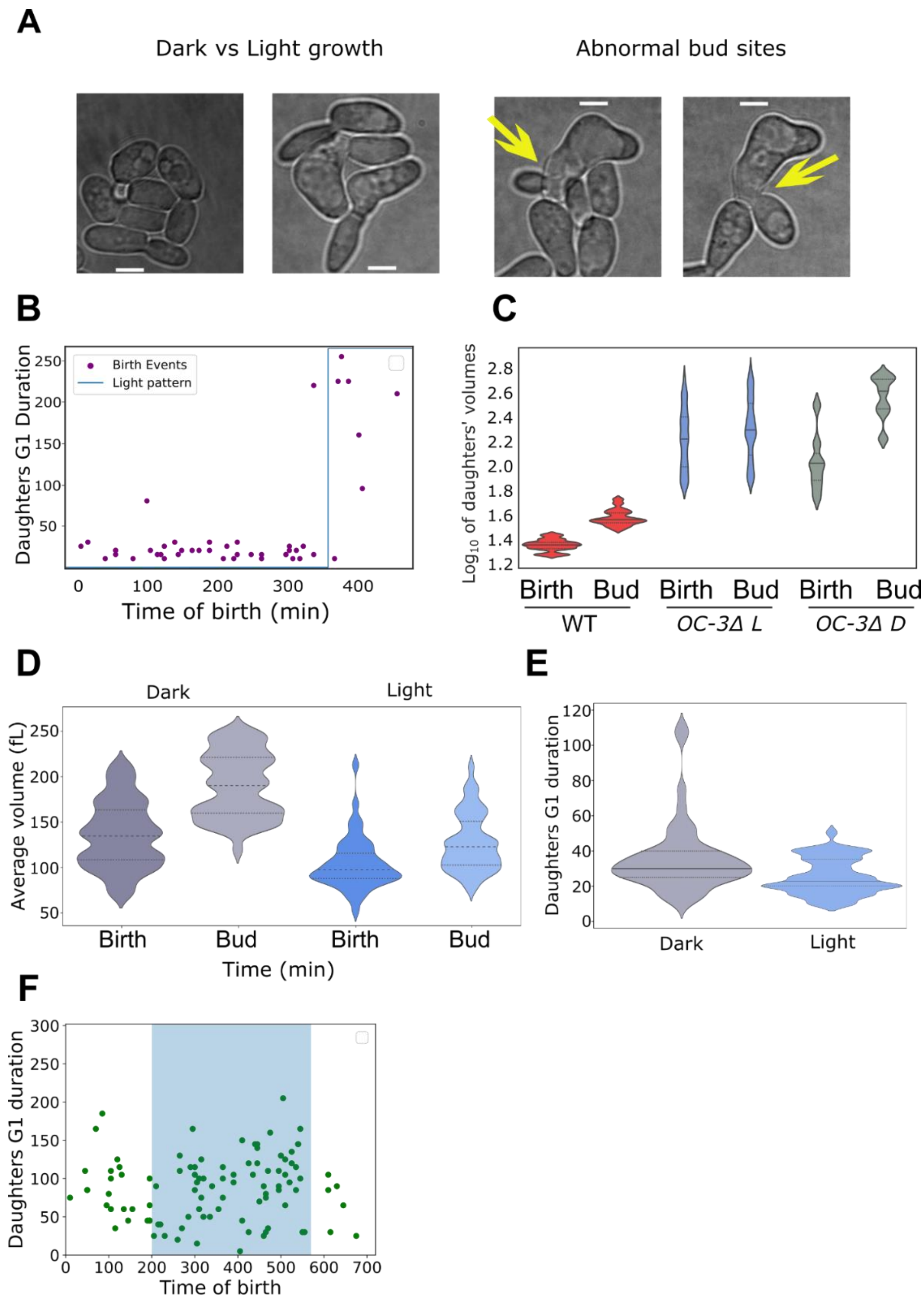

A
Dark vs Light growth
Abnormal bud sites
B
Daughters G1 Duration
Birth Events
Light pattern
Time of birth (min)
C
Log10 of daughters' volumes
Birth Bud
WT
OC-3Δ L
OC-3Δ D
D
Dark
Light
Average volume (fL)
Birth Bud Birth Bud
Time (min)
E
Daughters G1 duration
Dark
Light
F
Daughters G1 duration
Time of birth

Figure S3. pGAL1 OPTO-Cln2 cells and cells with an alternative CLN2 promoter truncation display phenotypic defects due to Cln2 overexpression**.** Related to Figure 2. **A)** Representative brightfield images of pGAL1 OPTO-Cln2 cells. Left image pair: comparison of cells grown in darkness vs light. Right image pair: examples of abnormal budding patterns during growth in blue light. **B)** Scatter plot of G1 durations in pGAL1 OPTO-Cln2 daughter cells during a transition from darkness to blue light. The x-axis denotes the birth moment of each tracked daughter, while the y-axis displays the corresponding G1 duration. Contrary to the expectation, daughter G1 durations are relatively short in darkness and increase massively when cells are shifted to light, indicating substantial leaky expression of Cln2 in darkness and Cln2 overexpression in the light. **C)** Volume distributions of daughter cells at birth and budding for WT (n = 17), pGAL1 OC-3Δ L (n = 43), pGAL1 OC-3Δ D (n = 45). Median and 25th and 75th percentiles are displayed with dashed lines. **D,E)** The OPTO-Cln2 construct (with SPO13pr) integrated at the X1 locus results in Cln2 overexpression, as suggested by the large volume of daughter cells during birth and budding in both light conditions (D) and the very short G1 duration in both darkness and light (E). **F)** An OPTO-Cln2 construct featuring a larger (1000 bp) truncation of the endogenous CLN2 promoter generates large heterogeneity in daughter G1 duration in both darkness and light conditions. Scatter plot of individual daughter G1 durations during transitions between darkness (white) and light (blue) conditions. Each dot corresponds to a single daughter cell, and its x-coordinate corresponds to the moment of daughter birth.

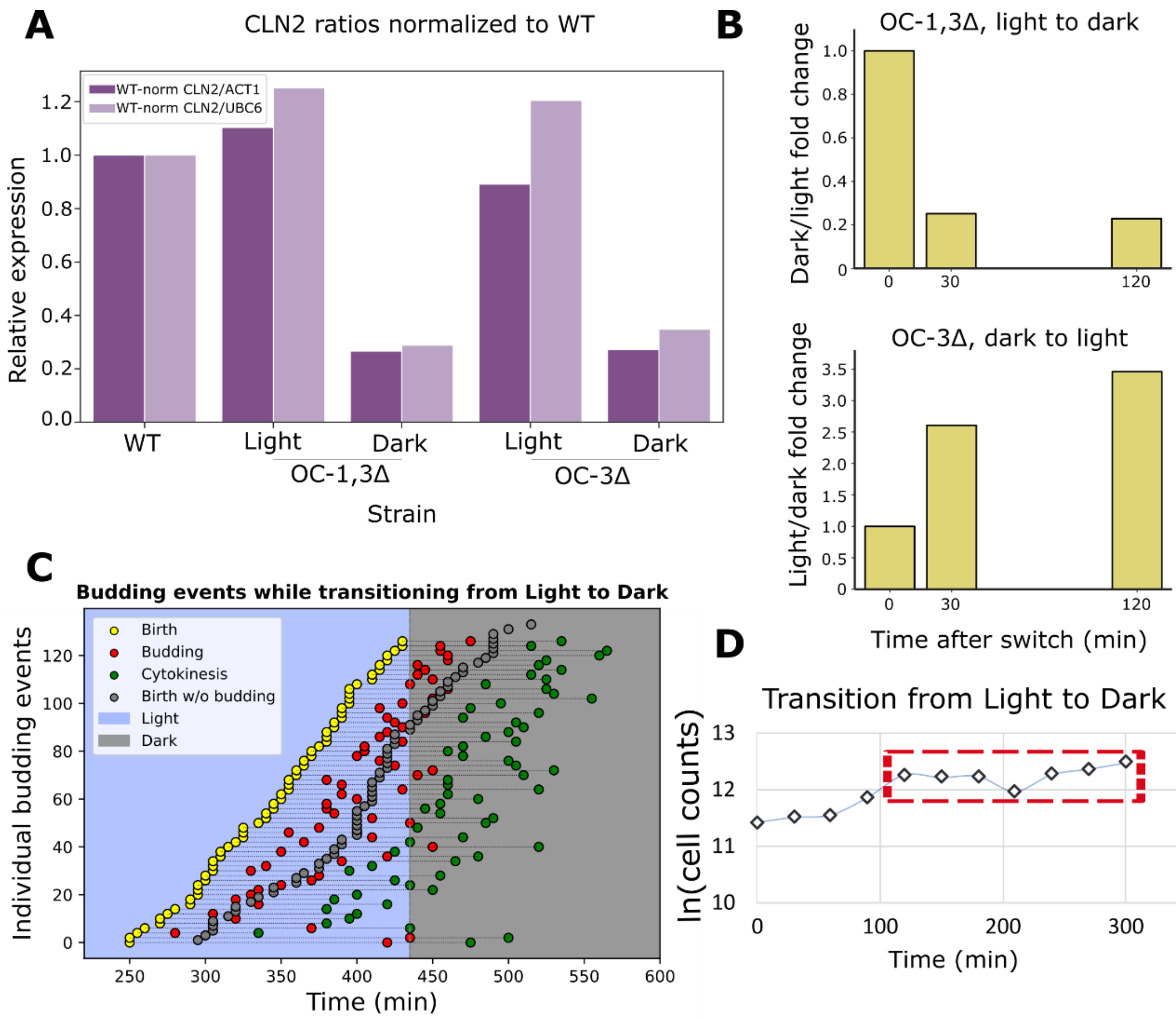

Figure S4: Quantification of Cln2 transcription in OPTO-Cln2 strains and proliferation dynamics in response to light transitions. Related to Figures 2 and 3. **A)** Comparison of Cln2 mRNA levels in darkness vs. light. Expression levels were determined as described in STAR Methods and are plotted relative to WT, using two different housekeeping genes (ACT1 and UBC6) for normalization. Data represent a single biological replicate. **B)** Cln2 mRNA levels during light transitions. The plots combine data from two experiments: i) the experiment of panel A, where mRNA levels were determined at time 0 and 120 min after light change. ii) a second experiment, where mRNA levels were determined at time 0 and 30 min after light change. For time points t=30 min and t=120 min, the relative expression level of Cln2 was determined with respect to Ubc6. Subsequently, the relative Cln2 expression level at t=30 min and t=120 min was divided by the corresponding relative expression level at t=0 to determine a fold change between the first and second light condition. Cln2 mRNA levels respond rapidly to light shifts, approaching near steady-state values within 30 minutes after a light transition. **C)** Cell cycle events of individual OC-1,3Δ daughters during a transition from blue light to darkness. Cells were tracked with microscopy for ~8h and the cell cycle events (birth, budding and cytokinesis) of the last cycle before the arrest were annotated for each daughter cell in the movie. This figure is an extension of Fig 3H, as it includes more daughters that budded, as well as daughters that did not bud until the end of the experiment (grey dots). **D)** Growth curve of OC-1,3Δ cells in YPD transitioning from a light-grown preculture that was shifted to darkness at t = 0. Post-G1 cells that encounter the light transition at t = 0 need to finish their ongoing cell cycle, which results in an increase in cell numbers ~100 min after the switch. From 100 min onwards the cell counts stabilize, indicating arrest of cell cycle progression.

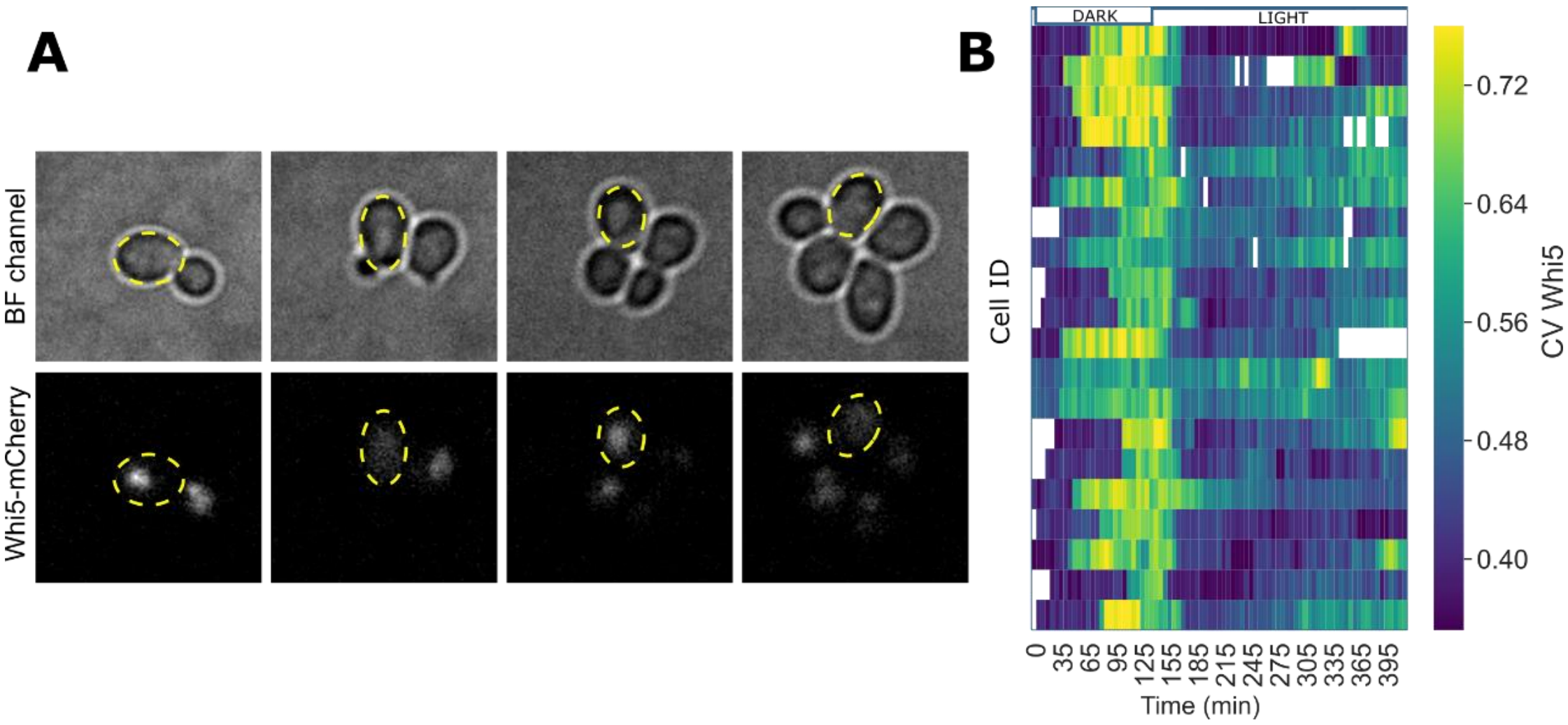

Figure S5. Quantification of Whi5 localization dynamics in OPTO-Cln2 *cln1Δcln3Δ* cells with fluorescence microscopy. Related to Figure 3. **A)** Representative brightfield and fluorescent microscopy images of Whi5-mCherry in OPTO-Cln2 *cln1Δcln3Δ* cells growing under constant illumination. Whi5 is nuclear in early G1 cells (first and third frame) but not in budded cells (second and fourth frames). **B)** Dynamics of Whi5 nuclear localization during light-dark-light transition**s**. The heatmap shows the coefficient of variation (CV) of Whi5 fluorescence over the cell area in individual OPTO-Cln2 *cln1Δcln3Δ* cells. Higher CV values correspond to nuclear Whi5 localization. Blank cells indicate time points where cells either were not present or could not be tracked. As indicated at the top, cells were grown in the light, shifted to darkness to induce transient arrest, and subsequently re-exposed to light.

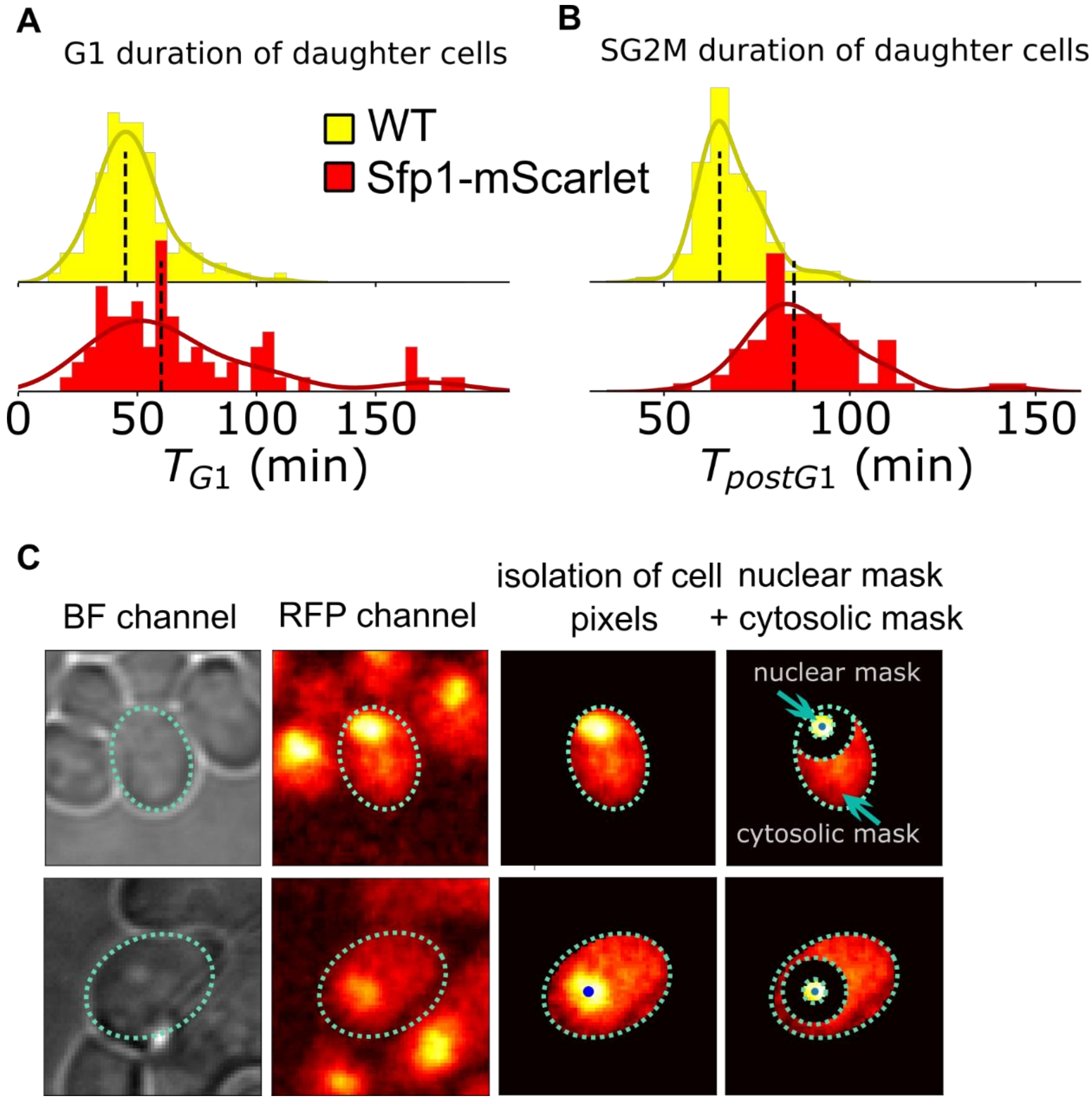

Figure S6. Cell cycle analysis of Sfp1-mScarlet-I cells and quantification of Sfp1-mScarlet-I nuclear localization. Related to Figure 4 and STAR Methods. **A, B)** Distributions of G1 and post-G1 durations of WT daughters and daughters expressing the Sfp1-mScarlet-I tag. Statistical comparisons, two-tailed Mann-Whitney test: WT_{G1} - $Sfp1_{G1}$, p = 0.07, |r| = 0.17, $WT_{post\text{-}G1}$ - $Sfp1_{post\text{-}G1}$, p = 8.5e-8, |r| = 0.5. **C)** Schematic representation of the pipeline used to analyze the N/C ratio of Sfp1-mScarlet-I. Cell segmentation generated with the BudJ plugin in the brightfield channel was used to define a cell mask in the RFP channel. The pixels within mask were processed via local thresholding (STAR Methods) to identify the brightest region in the image whose area does not exceed 30% of the total cell area (nuclear ROI). Nuclear Sfp1 intensity was calculated from a smaller region within the nuclear ROI. A larger mask with the same center as the nuclear ROI (black area) was defined to extend beyond the nucleus, and the remaining cell pixels were used to defined a cytosolic ROI. The Sfp1 N/C ratio was calculated by dividing the average intensity of the nuclear ROI by the average intensity of the cytosolic ROI.

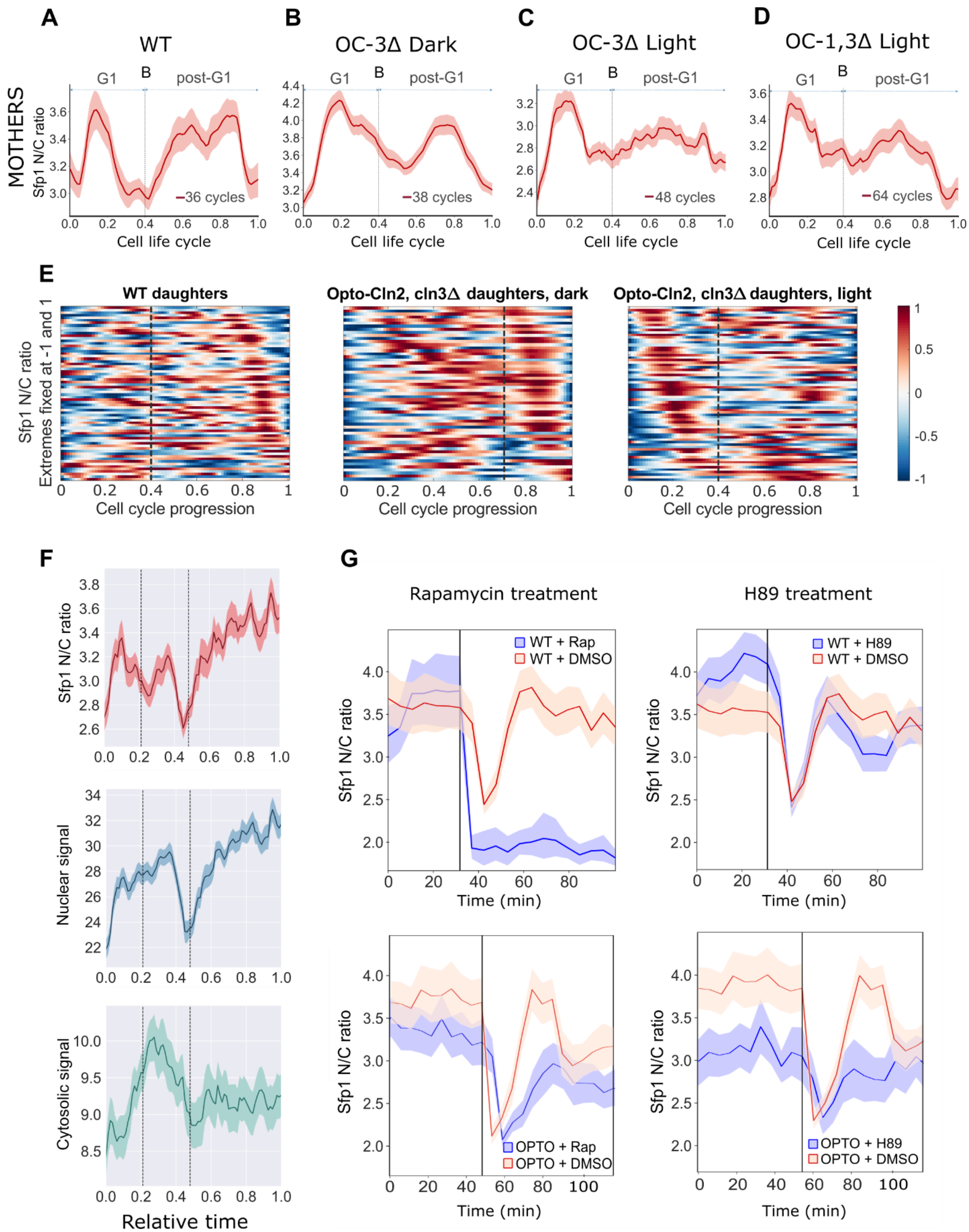
A
WT
B
OC-3Δ Dark
C
OC-3Δ Light
D
OC-1,3Δ Light
MOTHERS
Sfp1 N/C ratio
G1
B
post-G1
36 cycles
38 cycles
48 cycles
64 cycles
Cell life cycle
E
WT daughters
Opto-Cln2, cln3Δ daughters, dark
Opto-Cln2, cln3Δ daughters, light
Sfp1 N/C ratio
Extremes fixed at -1 and 1
Cell cycle progression
F
Sfp1 N/C ratio
Nuclear signal
Cytosolic signal
Relative time
G
Rapamycin treatment
H89 treatment
WT + Rap
WT + DMSO
WT + H89
WT + DMSO
OPTO + Rap
OPTO + DMSO
OPTO + H89
OPTO + DMSO
Sfp1 N/C ratio
Time (min)

Figure S7. Nuclear localization dynamics of Sfp1 during the cell cycle of OPTO-Cln2 strains and in response to drug treatments. Related to Figure 4. **A-D)** Nuclear localization dynamics of Sfp1 in mother cells of the strains shown in the panels. Cell cycle events (cytokinesis (leftmost point, corresponding to x=0), budding (B) and the following cytokinesis (rightmost point, corresponding to x=1)) were manually annotated in microscopy movies and were used to align the individual trajectories prior to averaging. In each plot, the placement of the budding point was based on the average ratio of G1 vs. total cell cycle duration of mothers of the corresponding strain. Shaded bands denote the 95% confidence interval for the mean. No major changes were observed in the G1 localization profile of Sfp1 in these strains. On the other hand, the attenuated localization peak of Sfp1 in G2 (especially in OC-3Δ and OC-1,3Δ) warrants further investigation. **E)** Heatmaps of Sfp1 nuclear-to-cytoplasmic (N/C) ratio dynamics in individual cell cycles of WT (n=70), OPTO-Cln2 *cln3Δ* cells grown under blue light (n=65) or in darkness (n=58). The heatmaps represent the single-cell data that were averaged in Figure 4C–E. To facilitate visualization, each trace was normalized such that its minimum and maximum values correspond to -1 and 1, respectively. Traces were aligned at birth, budding, and cytokinesis, as described in STAR Methods, with birth and cytokinesis corresponding to points 0 and 1 on the x-axis. The location of the budding event for each strain reflects the average fraction of G1 relative to the total division time across all measured cells. Because individual Sfp1 traces are noisy due to the absence of a nuclear marker, nuclear and cytosolic signals were smoothed using a LOESS filter with a smoothing parameter $\alpha = 0.35$. Finally, the N/C ratio traces were ordered according to the timing of either their first (OPTO-Cln2 *cln3Δ*, light) or last (OPTO-Cln2 *cln3Δ*, dark and WT) peak. **F)** Sfp1 dynamics in light-grown OPTO-Cln2 *cln1Δcln3Δ* mother cells following arrest in the dark. Single-cell trajectories were aligned at four points: the penultimate cytokinesis (x=0), the last budding (x=0.2), the last cytokinesis (x=0.45) and the last acquired time point (x=1, corresponding to ~160 min after the end of the last completed cell cycle). Top panel: average nuclear-to-cytoplasmic (N/C) ratio dynamics of Sfp1 (data of Fig. 4F, reproduced here to facilitate interpretation). Middle panel: average nuclear concentration dynamics of Sfp1 in the cells tracked for Fig. 4F. Bottom panel: average cytosolic concentration dynamics of Sfp1 in the same cells. The nuclear and cytosolic signals are expressed in the same arbitrary fluorescence units. **G)** Sfp1 localization dynamics in response to rapamycin (left panels) and H89 (right panels) treatment. Top row: treatment of wild-type cells carrying Sfp1-mScarlet-I. Bottom row: treatment of OPTO-Cln2 *cln1Δcln3Δ* cells following cell cycle arrest. The cells were transferred to darkness 2.5 h before drug addition, and imaging was started at t = 0. Vertical lines indicate the moment of inhibitor addition. Bands denote the 95% confidence interval of the mean. For each strain, the single DMSO control is repeated on two panels to facilitate comparisons.

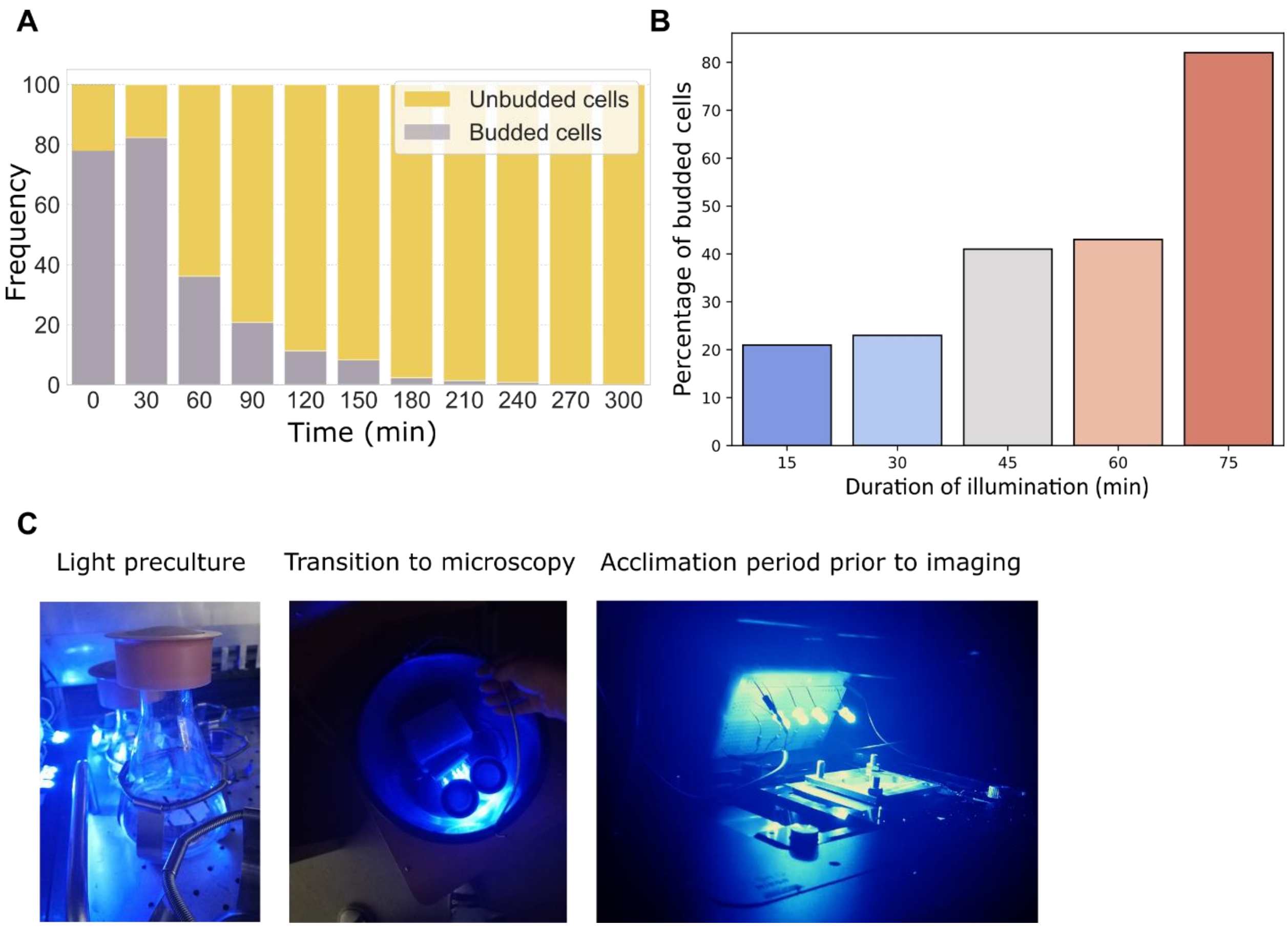

Figure S8. Budding cell counts of the OPTO-Cln2 *cln1Δcln3Δ* upon light transitions and illustration of the illumination setup. Related to Figure 5 and STAR Methods. **A)** Fraction of budded (grey) versus unbudded (yellow) OPTO-Cln2 *cln1Δcln3Δ* fixed cells following a transition from light to darkness at t = 0. **B)** Budding index measured 2 hours after illumination of OPTO-Cln2 *cln1Δcln3Δ* cells with light pulses of different durations. Cells were kept in darkness for ~2 h to arrest and were exposed to light for the indicated durations. The budding index was assessed 2 h after the light switch. **C)** Light illumination setup for liquid cultures of OC-1,3Δ cells in the shaking incubator (left), during their transfer to the microscopy room (middle) and during the acclimation period (1-2h) in the microscopy chamber before the start of an experiment.
